# A smartphone application toward detection of systolic hypertension in underserved populations

**DOI:** 10.1038/s41598-024-65269-w

**Published:** 2024-07-04

**Authors:** Cederick Landry, Vishaal Dhamotharan, Mark Freithaler, Alisse Hauspurg, Matthew F. Muldoon, Sanjeev G. Shroff, Anand Chandrasekhar, Ramakrishna Mukkamala

**Affiliations:** 1 Department of Bioengineering, University of Pittsburgh, 408 Benedum Hall, 3700 O’Hara Street, Pittsburgh, PA 15261 USA; 2 Department of Mechanical Engineering, University of Sherbrooke, Sherbrooke, QC Canada; 3 Department of Obstetrics, Gynecology and Reproductive Sciences, University of Pittsburgh School of Medicine, Pittsburgh, PA USA; 4 Magee Womens Hospital, University of Pittsburgh Medical Center, Pittsburgh, PA USA; 5 Division of Cardiology, Department of Medicine, University of Pittsburgh School of Medicine, Pittsburgh, PA USA; 6 Department of Electrical Engineering and Computer Science, Massachusetts Institute of Technology, Cambridge, MA USA; 7 Department of Anesthesiology and Perioperative Medicine, University of Pittsburgh, Pittsburgh, PA USA

**Keywords:** Risk factors, Engineering

## Abstract

High systolic blood pressure (BP) is the most important modifiable risk factor for cardiovascular disease. Managing systolic hypertension is especially difficult in underserved populations wherein access to cuff BP devices is limited. We showed that ubiquitous smartphones without force sensing can be converted into absolute pulse pressure (PP) monitors. The concept is for the user to perform guided thumb and hand maneuvers with the phone to induce cuff-like actuation and allow built-in sensors to make cuff-like measurements for computing PP. We developed an Android smartphone PP application. The ‘app’ could be learned by volunteers and yielded PP with total error < 8 mmHg against cuff PP (N = 24). We also analyzed a large population-level database comprising adults less than 65 years old to show that PP plus other basic information can detect systolic hypertension with ROC AUC of 0.9. The smartphone PP app could ultimately help reduce the burden of systolic hypertension in underserved populations and thus health disparities.

## Introduction

Although systolic hypertension is commonly defined as systolic blood pressure (BP) ≥ 130 or 140 mmHg, systolic BP ≥ 110–115 mmHg increases population risk for cardiovascular events^1^. Systolic hypertension, defined using the 110–115 mmHg threshold, afflicts more than 4 billion adults worldwide^1^. The prevalence has doubled over the last 30 years, and systolic hypertension has emerged as the leading modifiable risk factor contributing to global cardiovascular disease burden^1^. The bulk of the problem now resides in low- and middle-income countries where the prevalence of systolic hypertension is greater and an estimated 88% of deaths attributable to high systolic BP occur^2^. Secondary organ damage is also more pronounced in young and middle-aged adults and pregnant individuals in these countries compared to high-income countries^3^. One important reason for these global health inequalities is that underserved populations often have limited or no access to cuff devices for regularly checking BP. About 60% of hypertensives in underserved populations, in fact, are not even aware of their condition^3^.

Smartphones are being used far more than any other device and have significant uptake in low-income countries (e.g., 33% of adults report owning a smartphone in sub-Sahara Africa^4^). Converting smartphones into BP sensors could thus uniquely help in mitigating the burden of hypertension and reducing health disparities. Current smartphone BP measurement methods are based on machine learning analysis of photoplethysmography (PPG) waveforms obtained by placing a finger on the camera^5^ or processing of facial video^6,7^. Because PPG waveforms indicate blood volume oscillations, these ‘pulse wave analysis’ methods nominally require periodic calibrations with a cuff device to yield BP in mmHg units^8^. Pulse wave analysis is also not based on any generally accepted physiological principle, and extensively studied pulse wave analysis via consumer type wearables has not proven to offer significant value in BP measurement accuracy as yet^9,10^. Moreover, PPG waveforms obtained with smartphones may not be as reliable for pulse wave analysis as with wearables due to finger PPG contact pressure variations^11^ and even modest user motion during noncontact measurement^12,13^.

We considered the problem of measuring BP in mmHg units using only a smartphone. The challenge is that BP is basically a force, yet standard smartphones do not include force sensing. We were able to conceive a solution that invokes the proven automatic cuff device principle. The idea is for the user to perform guided thumb and hand maneuvers with the smartphone to induce actuation equivalent to cuff inflation/deflation and allow standard phone sensors to make measurements similar to a cuff transducer from the thumb artery. Absolute pulse pressure (PP = systolic BP–diastolic BP) can be computed from the measurements using a cuff algorithm.

In this article, we explain our smartphone PP concept. We then present an Android application (‘app’) for a popular smartphone to implement the concept. We follow with smartphone app usability results and accuracy results against a conventional, validated cuff device. We next show that measuring PP offers immense value in detecting systolic hypertension through machine learning analysis of a large population-level BP database. We lastly provide a thorough discussion of our study including related work, the various devices we developed and the many human studies we performed to explain how we arrived at the final app, and the next research steps. Ultimately, the app could uniquely help reduce the burden of systolic hypertension globally and particularly in underserved populations, as called for elsewhere^14^.

## Results

### Concept

Our smartphone concept invokes the oscillometric principle, which is employed by most automatic cuff devices^15^. Oscillometry is based on the sigmoidal blood volume-transmural pressure relationship of arteries, where transmural pressure is defined as the internal BP minus the external pressure of an artery. The principle generally involves sweeping over negative and positive transmural pressures of an artery to increase and then decrease its blood volume oscillation amplitude and then applying an algorithm to compute BP from the oscillation amplitude versus transmural pressure change function (‘oscillogram’). Conventionally, an inflatable cuff is used to vary the external (and thus transmural) pressure of the arm artery and measure the blood volume oscillations as the highpass filtered cuff pressure and the transmural pressure change as the lowpass filtered cuff pressure. In our smartphone concept, the user performs thumb and hand maneuvers to vary the transmural pressure of the thumb artery, while standard phone sensors are used to make cuff pressure-like measurements.

The hand maneuver is to slowly raise the hands with arms straight (elbows locked) from fully lowered to the anterior thighs to all the way above the head while holding the phone with both hands. This maneuver varies the internal BP in the thumb artery due to the weight of blood. For a typical arm length, the internal BP or ‘hydrostatic pressure swing’ is about ± 45 mmHg relative to heart level. For a mean thumb BP of 80 mmHg, the transmural pressure would range from about 35 to 125 mmHg. However, both positive and negative transmural pressure regimes are needed to attain a complete (‘inverted U’ shape) oscillogram for accurate BP computation. The thumb maneuver is thus to increase the thumb contact pressure on the phone (i.e., the external pressure of the target artery) to roughly around the mean BP and then maintain this contact pressure during the hand raising so that the transmural pressure range is about ± 45 mmHg. Standard smartphone sensors are also leveraged along with other phone features to guide the user in performing the two maneuvers.

**Figure 1:**
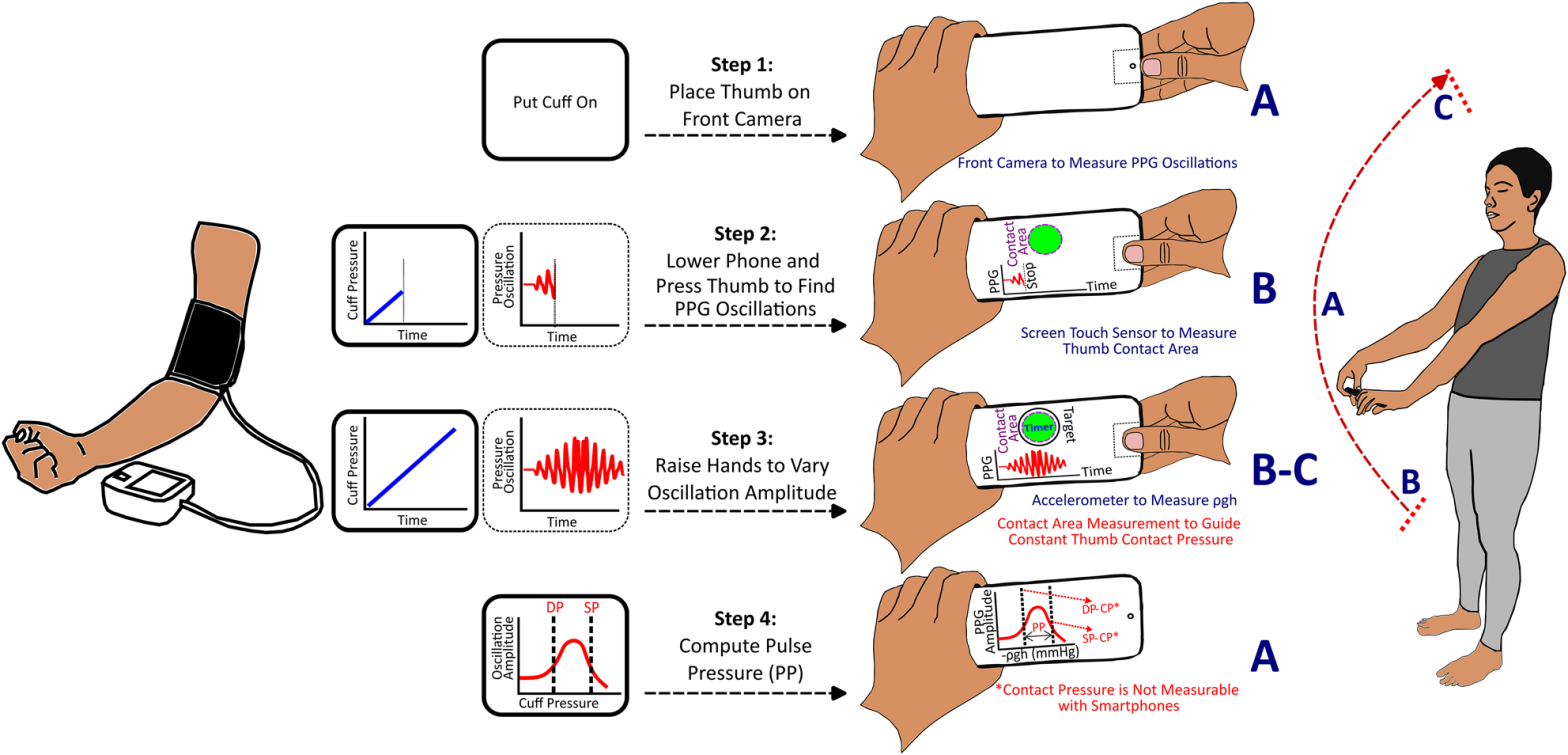
Smartphone pulse pressure (PP) concept in relation to the analogous steps of automatic cuff blood pressure (BP) measurement. The user performs thumb pressing and hand raising with the phone to sweep the transmural pressure of the thumb artery, essentially replicating cuff inflation/deflation, and standard phone sensors are used to make cuff-like measurements and guide the user in performing the maneuvers. PP is computed from the measurements via a cuff algorithm. PPG is photoplethysmography and ρgh, hydrostatic pressure change via hand raising (where ρ is blood density, g is gravity constant, and h is vertical height of phone relative to heart).

Figure 1 illustrates the complete smartphone concept and how it parallels the four steps of classic arm cuff BP measurement. In step 1, the user holds the phone with one hand and places the thumb of the other hand on the front camera and screen with the other fingers on the back side of the phone. The app guides the thumb placement by displaying a rectangular box on the screen and measures the PPG waveform via the front camera. In step 2, the user fully lowers their hands and presses their thumb uniformly and slowly against the phone to increase the external pressure of the underlying transverse palmar arch artery to reach roughly around the mean BP as indicated by appreciable blood volume oscillations. The app guides this thumb maneuver by displaying and analyzing the PPG waveform and displaying the thumb contact area on the screen via the screen capacitive touch sensor as well as the target area. In step 3, the user raises their hands with arms fully extended and wrist-hand angle locked to all the way above their head in continuous motion over 20–40 s while maintaining the thumb contact area. The app guides this maneuver by displaying a timer and the current and target thumb contact areas while measuring the change in hydrostatic pressure (ρgh, where ρ is the known blood density, g is the gravity constant, and h is the vertical height of the phone relative to the heart) in mmHg units via the accelerometer and arm length. In step 4, the app constructs the oscillation amplitude versus ρgh function. This function is the oscillogram shifted to the left by the unknown thumb contact pressure. As a result, systolic BP or diastolic BP cannot be ascertained. However, PP is indicated by the width of the shifted oscillogram.

### Smartphone PP application (‘app’)

We developed an Android app for the Samsung Galaxy S21 to implement the smartphone PP concept. Android was chosen as it is the most popular mobile platform worldwide, but other operating systems as well as other phones could have been used.

The app uses the red channel of the front camera along with ambient light and screen light (bright setting) to measure the PPG waveform. Due to the smartphone orientation during the hand raise (see Fig. 1), the app uses the z-axis (front-to-back) channel of the accelerometer to measure the hydrostatic pressure change, ρgh. According to basic trigonometry, the channel output of +1 (fully lowered phone) to −1 (fully raised phone) is multiplied by the blood density ρ and the user arm length to yield ρgh in mmHg units. The arm length can be measured or estimated based on the user height. The app uses the x-coordinate of the screen touch centroid to represent the thumb contact area.

**Figure 2:**
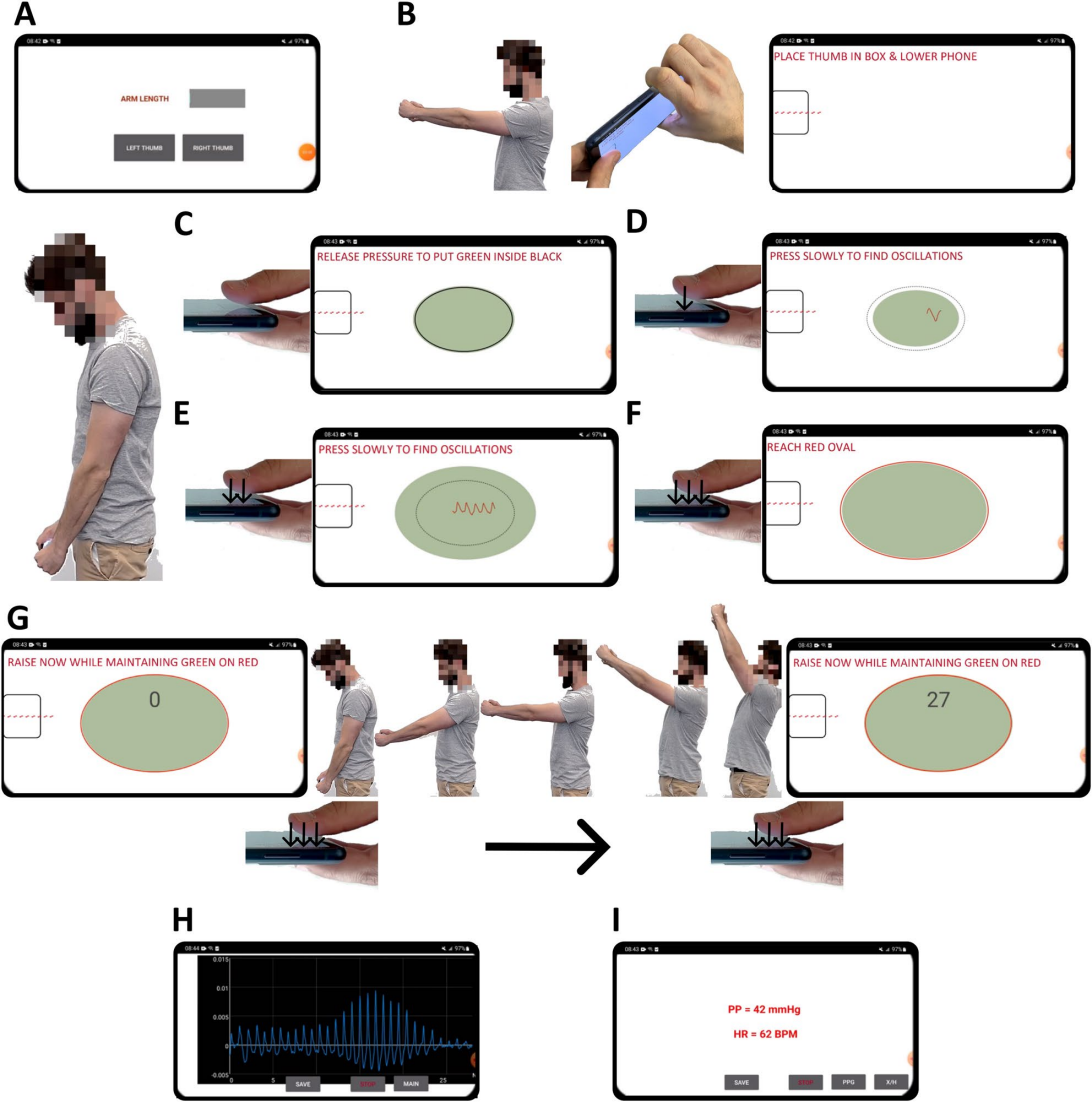
Smartphone android application (‘app’) to measure PP. (**A**) A one-time initialization screen is shown for the user to enter their arm length and thumb for pressing. (**B**) A rectangular box follows to guide the user in placing their thumb over the front camera for measuring the thumb PPG waveform and on the screen. Once the user lowers the phone, (**C**) a green shaded oval is displayed whose size reflects the real-time thumb contact area via the screen touch sensor along with a target (black) oval to ensure minimal initial thumb contact. (**D**) Once the user brings the green shaded oval within the target oval through a light press, the PPG waveform (red trace) appears as noise. (**E**) The user presses slowly to increase the green shaded oval size until blood volume oscillations appear. (**F**) The oscillations are automatically detected, and a red oval is displayed whose size reflects the target thumb contact area. Once the user presses further to achieve the target, (**G**) a timer is presented for the user to perform hand raising in 20–40 s while maintaining their thumb contact area, (**H**) The measured blood volume oscillations versus time are displayed. (**I**) The computed PP via the derivative algorithm (see Fig. 5) and heart rate (HR) are finally presented. The subject in the figure provided informed consent to publish the images.

Figure 2 illustrates the app including its guidance and measurements. A one-time initialization screen appears for the user to enter their arm length and thumb for pressing (Fig. 2A). A standard rectangular box follows near the front camera to guide the user in placing their thumb on the phone (Fig. 2B). For users with noncongruent thumb sizes, a personalized rectangular box can be defined through extension of the one-time initialization as described in^17^. The app prompts the user to place the thumb in the box and fully lower the phone. A green shaded oval whose size reflects the real-time thumb contact area then appears on the app along with a target (black) oval (Fig. 2C). The size of the target oval ensures minimal initial thumb contact. Once the user brings the green oval within the target oval through a light press, the PPG waveform (red trace) appears (Fig. 2D). Because of the minimal thumb contact pressure, the waveform will appear as noise. The user presses slowly until blood volume oscillations appear (Fig. 2E). The app detects the oscillations and then displays a red oval whose size reflects a fixed percentage increase in the current thumb area (Fig. 2F). The user continues to press until the green oval reaches the red target. The app thereafter displays a timer (Fig. 2G). The user raises their hands in continuous motion over 20–40 s while maintaining the green oval aligned on top of the red one. The app displays the measured blood volume oscillations versus time (Fig. 2H). The app computes PP in mmHg units by applying the classic derivative algorithm^18^ to the oscillation amplitude versus ρgh function as well as the heart rate (HR). The app displays both PP and HR (Fig. 2I).

If the blood volume oscillation amplitude does not show an inverted U pattern, the blood volume oscillations are corrupted by noise, the thumb contact area changes significantly over time (which indicates a change in thumb contact pressure), or ρgh is not largely monotonic (which indicates a change in wrist-hand angle), the measurement is not valid and the user can try again. Further details and a video demonstration of the app are available (Supplementary Information 1, Supplementary Video 1).

### Usability and accuracy results

We tested the app for usability and accuracy in volunteers under IRB approval. Twenty-four volunteers (54% female; 36 ± 11 years; 168 ± 6 cm; 75 ± 10 kg) participated. Eight participants were experienced users, and the other 16 participants were inexperienced users.

The inexperienced users participated on a practice day and then a measurement day, whereas the experienced users only participated on the measurement day. On the practice day, each user learned how to use the app and then performed trials with the app until they made three valid measurements. On the measurement day, each user performed trials with the app until they likewise made three valid measurements. We obtained conventional automatic arm cuff BP measurements via a validated device as reference, and two of the experienced users also performed mental arithmetic to increase their PP.

**Figure 3:**
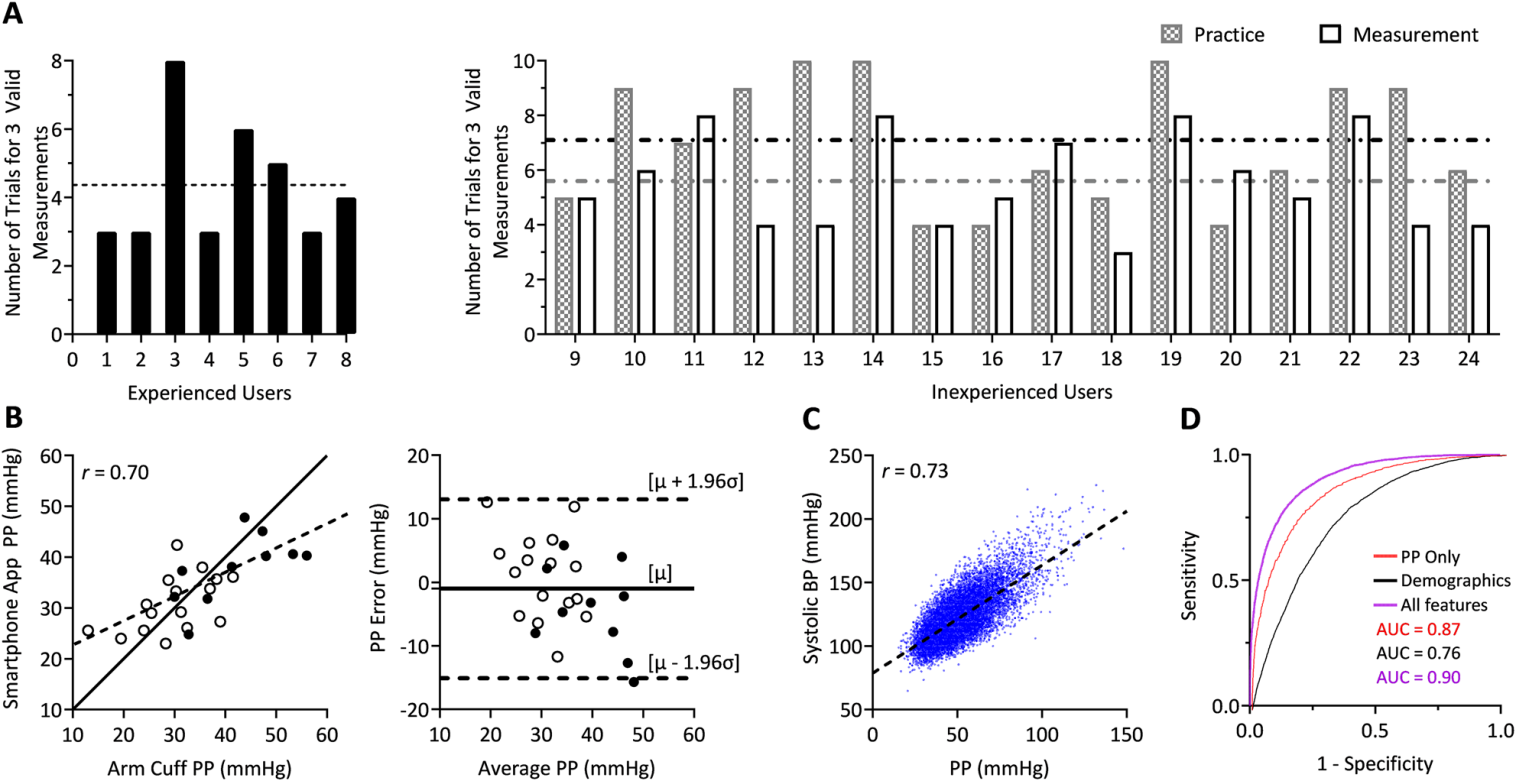
Study results: usability and accuracy of the smartphone PP app (N = 24) and systolic hypertension detection based on PP in the NHANES database^16^. (**A**) Number of attempts in making three valid smartphone measurements (see text for definition) for each experienced user on the measurement day and each inexperienced user on the practice and measurement days. Horizontal lines indicate mean values. (**B**) Correlation and bland–altman plots of the smartphone PP measurements versus reference automatic arm cuff PP measurements. White dots indicate inexperienced users, while black dots indicate experienced users. Solid line is identity line/bias error; dashed line, line of best fit/limits-of-agreement; r, correlation coefficient; and µ and σ, bias error (mean of errors) and precision error (standard deviation of errors), respectively. (**C**) Correlation plot between average systolic BP and average PP in the NHANES database (N = 19,212). (**D**) Receiver operating characteristic (ROC) curves for three neural networks to detect systolic hypertension (systolic BP ≥ 130 mmHg) from average PP only; demographics along with smoking history (demographics); and average PP, heart rate, demographics, and smoking history (all features) (N = 5,764). AUC is area under the curve.

Figure 3A shows the usability results per participant. The inexperienced users performed on average six to seven trials to obtain three valid measurements for a 47% success rate on the practice day and increased their average success rate to 60% on the measurement day. The experienced users achieved an average success rate of 80% in obtaining three valid measurements. Figure 3B shows correlation and Bland–Altman plots of the average smartphone PP over the valid measurements versus the average arm cuff PP. The smartphone app yielded a correlation coefficient (r) of 0.70 and a bias error (µ) and precision error (σ) of −1.0 and 7.2 mmHg, respectively, over about a 40 mmHg reference PP range. The PP accuracy appeared similar for the inexperienced and experienced users (compare red and black datapoints in Fig. 3B).

### Systolic hypertension detection based on PP

PP is known to correlate well with systolic BP. To illustrate the value of PP in detecting systolic hypertension, we analyzed the National Health and Nutrition Examination Survey (NHANES) database from 2007 to 2016^16^. We included the triplicate auscultatory BP measurements, HR, demographics (age, sex, race, and body mass index), and smoking history in our analysis. We excluded participants with missing information and above 65 years old, as our target population for the app is adults less than 65 years old. A total of 19,212 participant records remained. Figure 3C shows a plot of average systolic BP versus average PP. We trained and tested three neural networks to detect systolic hypertension (systolic BP ≥ 130 mmHg) from average PP alone, only demographics and smoking history, and average PP, HR, demographics, and smoking history. We employed a 70–30% training-testing data split. Figure 3D shows the receiver operating characteristic (ROC) curves for each network in detecting systolic hypertension in the testing data. The results show that PP along with other readily available information can effectively detect systolic hypertension with a ROC area under the curve (AUC) of 0.9.

To initially assess the impact of inevitable PP measurement error in the detection of systolic hypertension, we added Gaussian random noise to the PP measurements in the testing dataset with mean and standard deviation of −1.0 and 7.2 mmHg (i.e., the bias and precision error of the smartphone PP app shown in Fig. 3B) and then tested the already trained neural network on these data. We repeated this process for 100 different noise realizations. The ROC AUC decreased modestly from 0.9 to 0.87 ± 0.003.

## Discussion

Previously, we proposed the ‘oscillometric finger pressing method’ for BP monitoring via a smartphone^19^. The user presses their fingertip against the smartphone held at heart level to steadily increase the external pressure of the underlying artery, while a custom PPG-force sensor unit attached to the phone measures the resulting variable amplitude blood volume oscillations and applied finger pressure. The phone also visually guides the amount of finger pressure that the user applies over time and computes systolic and diastolic BP from the constructed oscillogram. We later showed that this method could be implemented as an iPhone X app that leverages the front camera to measure the PPG waveform and the 3D touch sensor under the adjacent screen to measure finger force^17^. Although exquisite, the 3D touch sensor was not designed for BP measurement and was thus suboptimal (e.g., the finger force could saturate prematurely for higher BP or larger fingers). Moreover, the finger contact area could only be estimated, which introduced significant error in the BP measurement. Apple discontinued 3D touch in subsequent versions of the iPhone (11 and onwards) anyhow, and few, if any, phones today include fine force sensing.

The smartphone app that we have introduced herein circumvents the need for a force sensor by varying the internal BP via hand raising and accurately measuring the hydrostatic pressure change with the accelerometer. In conventional oscillometry, the external pressure of the artery is swept. The clever idea of implementing oscillometry via internal BP changes induced by hand raising was proposed by Shaltis et al.^20–22^. These investigators built a finger worn ring comprising a PPG sensor, accelerometer, and force sensor. This ring could potentially measure systolic and diastolic BP from the function relating the blood volume oscillation amplitude to the difference between the contact pressure of the ring on the finger and the hydrostatic pressure change (ρgh, via the accelerometer). However, the investigators did not address how to attain and maintain a finger contact pressure around the mean BP to obtain a full oscillogram. This step is not only an enabling one but also nontrivial, because mean BP is essentially what is sought for measurement. Furthermore, they only published results from five volunteers, so the ‘oscillometric hand raising method’ has remained mostly theoretical until now.

Our overall idea was to translate the oscillometric hand raising method for implementation as a smartphone app (see Fig. 1). We needed to conceive two key innovations to realize the app. Firstly, we devised a procedure to determine and maintain the proper thumb contact pressure on the phone using combined guidance from the blood volume oscillations via the front camera and the adjacent screen touch sensor. Secondly, we eliminated the need for a force sensor by using the screen touch sensor as a surrogate and focusing on PP. We also showed that PP plus other readily available information can effectively detect systolic hypertension with ROC AUC of 0.9 by training and testing neural networks on a population-level database (see Figs. 3C, D). (PP with the other information could detect general hypertension (systolic BP ≥ 130 mmHg or diastolic BP ≥ 80 mmHg) with ROC AUC of 0.81 (not shown).)

**Figure 4:**
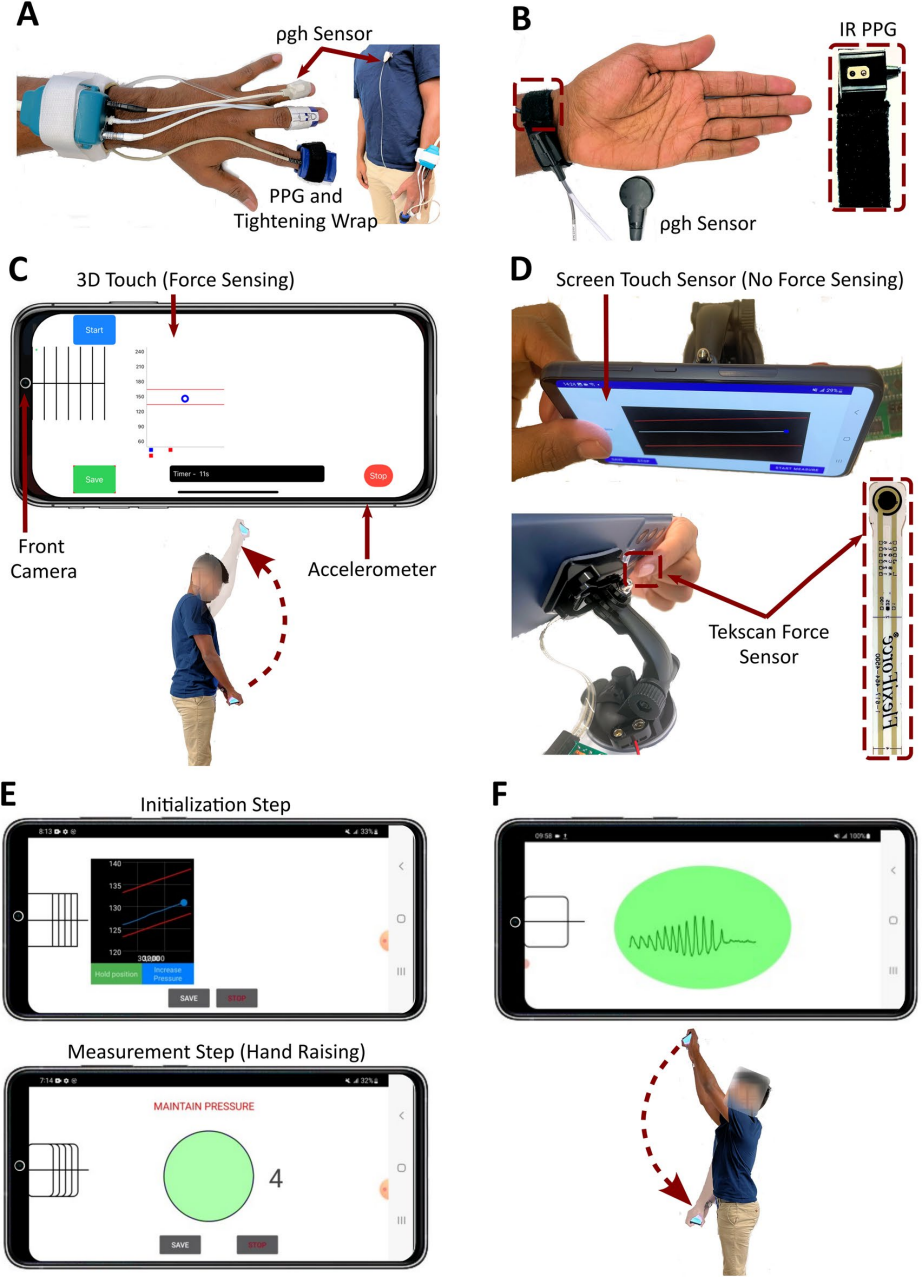
Devices that were initially developed and studied in volunteers to arrive at the final app. (A) ‘Ideal sensors’ to implement the oscillometric hand raising/lowering method for measuring PP (Supplementary Information 2). (**B**) Wrist device to implement the PP measurement method (Supplementary Information 3). IR is infrared. **(C)** iPhone X app to implement the smartphone PP concept via ‘ideal guidance’ with a direct contact force measurement (Supplementary Information 4). (**D**) System to investigate screen touch parameters as a surrogate of contact pressure (Supplementary Information 5). (**E**) First version of an Android app for a conventional smartphone without force sensing involving an intended-to-be infrequent initialization step to determine the target thumb contact area followed by a measurement step (Supplementary Information 6). (**F**) The second version of the smartphone Android app involving hand lowering actuation (Supplementary Information 7). The subject in the figure provided informed consent to publish the images.

While we came up with the main ideas prior to embarking on the study, it turned out to be a long journey to reach the final app and results. We ended up developing a number of different devices, as shown in Fig. 4, and studying the devices in volunteers with arm cuff PP as reference. Overall, we performed around 100 human studies under IRB approval in this work. We describe these initial studies below to importantly explain why the final app is the way it is.

We started with ‘ideal sensors’ to see if the oscillometric hand raising method even works (Fig. 4A, Supp. Mat. 3). We were concerned about the possibility of changes in smooth muscle contraction when raising the entire arm, which would violate the oscillometry assumption that the sigmoidal arterial blood volume-transmural pressure relationship is invariant during the measurement. Using a transmissive-mode infrared PPG clip with a Velcro tightening wrap and a fluid-filled tube-manometer system to directly measure ρgh, we found that PP could be measured fairly well via slow, continuous (rather than incremental) hand raising or lowering. This initial study convinced us that the oscillometric hand raising/lowering method is worthy of pursuit.

We studied wrist devices as an alternate way to implement the method (Fig. 4B, Supp. Mat. 4). While such devices are far less used than smartphones, the PP measurement would be more convenient in that the user would not have to maintain the contact pressure on the sensor. We found that devices embedded with PPG and direct ρgh sensors could measure PP well when the PPG sensor was placed over the radial artery but not the back of the wrist (similar to many consumer devices). However, locating the radial artery was too difficult. We concluded that smartwatches and fitness trackers currently in use cannot be converted into absolute BP sensors.

We began our study of smartphones using an iPhone X (Fig. 4C, Supp. Mat. 5). We could thus assess the feasibility of the user in maintaining the contact pressure during the hand raising via ‘ideal guidance’ with a direct contact force measurement (3D touch). We also initially explored the rear camera with flash for best measurement of the PPG waveform (from the index fingertip). However, smartphones have different rear camera configurations, so the app would have to be substantially modified for each phone. The front camera, on the other hand, is relatively standard and became our focus for greater generality. We found that users could indeed maintain the contact force during hand raising and lowering. We also determined that visual feedback of thumb contact was necessary, as there is a general tendency for the user to increase/decrease thumb contact pressure when performing hand raising/lowering due to the weight of the phone. We importantly found that holding the phone firmly with the supporting hand could mitigate this tendency.

We then studied the screen touch sensor as a surrogate for a force sensor (Fig. 4D, Supp. Mat. 6). We attached a force sensor to the back of a Samsung Galaxy S21 smartphone to measure the screen touch parameters (major and minor radii and x- and y-centroids) and thumb contact pressure simultaneously during thumb pressing. As expected, we found that the function relating each screen touch parameter to the thumb contact pressure increased progressively and then plateaued. However, we importantly discovered that the x-centroid plateaued last (i.e., it exhibited the greatest sensitivity at higher thumb contact pressures) and was least spurious of the touch parameters. We concluded that the touch x-centroid represents the thumb contact area best and could potentially guide the determination and maintenance of the thumb contact pressure.

We thereafter developed the first version of an Android app for the Samsung Galaxy S21 smartphone (Fig. 4E, Supp. Mat. 7). The app uses the front camera to measure the thumb PPG waveform, the z-axis accelerometer channel to measure ρgh, and the touch x-centroid sensor to measure the thumb contact area. In an ‘initialization step’, the user holds the device at heart level and slowly presses their thumb to increase and then decrease the blood volume oscillations while guided by the real-time and linear target thumb contact areas. The thumb contact area at the maximum oscillation amplitude (which occurs around the mean thumb BP) is selected as the target area. Then, in a ‘measurement step’, the user presses their thumb to reach this target area with the phone fully lowered and raises their hands in continuous motion over 20–40 s while maintaining the thumb contact area as guided additionally by a timer. The idea was that the initialization step need only be repeated when complete oscillograms are not obtained as a result of large BP changes in a user. The app also displays multiple rectangular boxes to determine the thumb position on the front camera and screen that yields the largest maximum oscillation for each user. The app could measure PP reasonably well via the same rectangular box for a number of users. However, it was too difficult to use. Firstly, linear thumb pressing was not easy, as the thumb contact area for guidance is not indicative of thumb contact pressure at high pressures. Secondly, the target thumb contact area was often not suitable due to variations in thumb positioning and pressing angle per measurement. We concluded that the target thumb contact area must be determined for each PP measurement and that the blood volume oscillation amplitude, which is determined by the thumb contact pressure when the device is at a fixed vertical level, should be used as a guide.

We thus developed a second version of the Android smartphone app (Fig. 4F, Supp. Mat. 8). The user holds the phone above their head and presses their thumb on the front camera and screen until the blood volume oscillations disappear. The app then displays the current thumb contact area as the target. The user thereafter lowers the phone in continuous motion while maintaining the thumb contact area on the target. Volunteers could achieve the arterial occlusion. However, very narrow oscillograms often resulted despite apparent maintenance of the thumb contact area over time. Based on our earlier studies, we reasoned that the thumb contact pressure must have decreased during hand lowering (which would increase the transmural pressure prematurely and narrow the oscillogram) but was not picked up by the thumb contact area measurement. We believe that thumb tissue viscoelasticity was the culprit (i.e., thumb contact area decreases slowly in response to a step decrease in thumb contact pressure). We concluded that the screen touch sensor is only a useful guide for hand raising in which the tendency is to increase the contact pressure.

**Figure 5:**
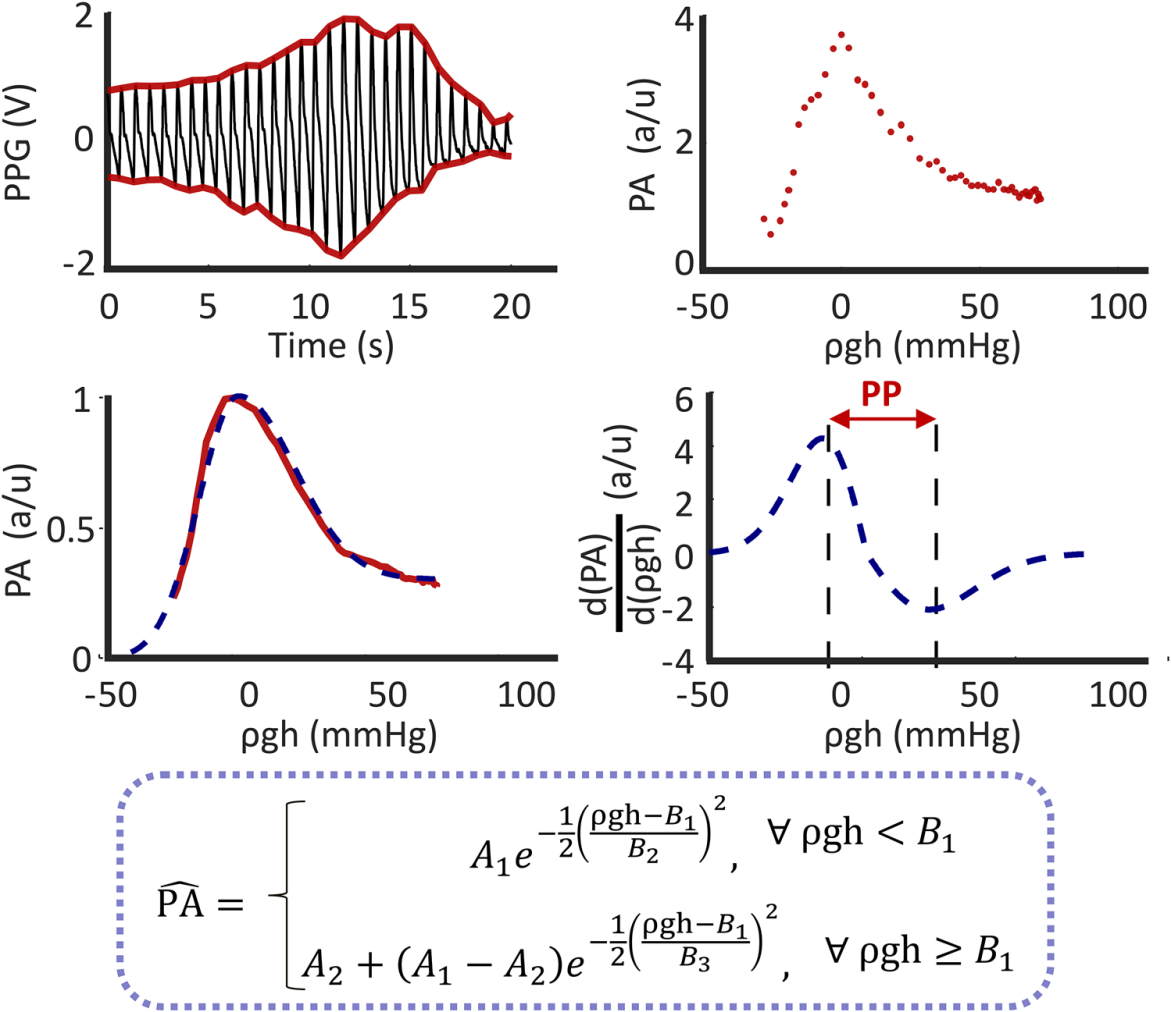
PP computation algorithm of the smartphone app. The beats of the PPG waveform are detected. The peak-to-peak PPG amplitudes (PA), normalized to a maximum of unity, are plotted against the corresponding ρgh values to produce a discrete oscillogram. The oscillogram is smoothed and fitted to a function with adjustable parameters (${A}_{n}$ and ${B}_{n}$) to yield a continuous oscillogram. The derivative of this oscillogram is taken, and PP is detected as the width between its maximum and minimum^18^. HR is also determined from the beat detections.

We developed a third and final Android smartphone app by revisiting hand raising (see Fig. 2). The key challenge was to determine the target thumb contact area when the hands are in the initial fully lowered position. The reason is that confounding blood volume oscillations appear at low contact pressure due to arterioles and other smaller vessels in the thumb (see Fig. 5)^18^. So, simply having the user press their thumb on the phone until oscillations appear may often yield a target area that is too small to abolish the oscillations when the hands are fully raised. We ended up conceiving a heuristic procedure to overcome this challenge. The user presses on the phone until blood volume oscillations appear. The phone automatically detects the oscillations and then displays a target thumb contact area that is a fixed percentage higher than the current area. The user further presses until the current thumb contact area reaches the target. The user then slowly raises their hands while maintaining the thumb contact area on the target. The app computes PP from the function relating the blood volume oscillation amplitude via the front camera to ρgh via the z-axis accelerometer channel and the arm length (‘shifted oscillogram’). While skin tone is not a major concern for the app due to PPG measurement from the palmar side of the thumb where melanin is less abundant, cold environments do compromise visible-light PPG waveform quality.

We tested the app for usability in 24 participants (see Fig. 3A). We first explained and demonstrated how to use the app. An accompanying video tutorial could alternatively be included (Supp. Mat. 2). The procedures to be learned, which are not guided by the app, are holding the phone tightly with the supporting hand, pressing the thumb straight down, and pressing at a consistent pace to transition from a noisy signal to blood volume oscillations, and locking the wrist-hand angle during the hand raise. Of these procedures, the steepest learning curve was pressing the thumb to elicit blood volume oscillations at relatively low thumb contact. Yet, after 6–7 practice trials, new users were able to make valid measurements with 60% success rate. Most users could start making valid measurements after 3–4 practice trials. The success rate reached 80% for experienced users and may increase further with regular app usage. We hypothesize that regular app usage may also minimize any changes in the user’s PP that could potentially occur due to the act of performing the thumb and hand maneuvers, which could increase stress for example.

The heuristic procedure for determining the target thumb contact area was effective in 22 of the 24 participants. In the remaining two participants, the oscillograms did not show an ascending limb despite proper app usage. The fixed percentage increase was simply lowered by half with the app, and complete oscillograms were then readily obtained. Conversely, if the oscillogram does not show a descending limb, then the fixed percentage increase could be raised.

We also tested the app for accuracy against a validated automatic arm cuff device in the same participants (see Fig. 3B). The PP via the app yielded r = 0.70 and an overall error < 8 mmHg against the reference PP over a 40 mmHg PP range. This level of agreement was similar to that obtained with the ideal sensors (Supp. Mat. 3), thereby further indicating that the users were able to effectively perform the thumb and hand maneuvers.

There are several sources of error. Firstly, we used the classic derivative algorithm to compute PP. This algorithm can amplify noise and underestimate PP in the thumb artery, especially in the higher PP range, due to small artery viscoelasticity (see Fig. 3B)^23^. However, it is possible to overcome viscoelasticity, while avoiding derivatives, to accurately compute PP^23^. Secondly, the reference device that we used measures arm PP. Thumb PP may be variably higher than arm PP due to arterial wave reflection^24^. We did employ a volume-clamp cuff device to obtain a finger BP waveform. However, as we previously found^18^, the thumb PP correlated better with the more important arm cuff PP. Thirdly, we used a less reliable automatic cuff device as reference.

Another limitation of our study was that few participants with Stage 2 hypertension were included. However, we expect that our smartphone PP app can be effective in hypertension. In our studies of the Android app, we observed that the effective thumb contact area range was about 75–95% of the maximum thumb contact area of the users and that the target thumb contact area corresponded on average to 85% of the maximum. Based on an exponential function for relating touch x-centroid to the thumb contact pressure with average parameter values (Supp. Mat. 6), a target thumb contact area of 90% of the maximum would correspond to a thumb contact pressure of 125 mmHg. Since mean thumb BP is about 10 mmHg lower than mean arm BP^24^, the app may thus be applicable in hypertensive users with mean BP of around 135 mmHg (e.g., 180/110 mmHg). Although the app may not be effective in users with higher mean BP or, since the BP swing via hand raising is typically about 80 mmHg, very wide PP (e.g., 120 mmHg), it is designed for people < 65 years in which extreme BP levels are even less common.

The major limitation of the smartphone PP app is indeed that it is not applicable to much of the elderly population. It is well known that the elderly are most afflicted by systolic hypertension. However, while regular BP measurement in the elderly is important to help with hypertension control, detection of hypertension in this population may not be vital. The reason is that virtually every person older than ~ 70 years may be sensibly assumed to be at a systolic BP level of increased risk for cardiovascular events^25^. So, from a hypertension detection perspective, the younger and middle-aged populations may actually be most relevant.

Future research is needed to bring the smartphone PP app to practice. Firstly, an accurate PP algorithm must be developed. We suggest to collect training data comprising ideal sensor measurements and manual auscultatory cuff BP measurements as reference from a large cohort of participants of diverse BP levels. The assumption here is that user error is random and could not be compensated for algorithmically. Secondly, this app must be tested rigorously for accuracy and usability in the field. Thirdly, the app should be tested with various smartphones due to differences in functionality (e.g., maximal screen brightness). It may also be worthwhile to explore obtaining diastolic BP information with the app. For example, if the target thumb contact area is low relative to the maximum thumb contact area of the user, then it may be reasonable to conclude low diastolic BP. That said, it is high systolic BP that is the most important modifiable risk factor^26^.

Following successful future research, the smartphone PP app (Android, iOS, and other versions) could be used by anyone who has or knows someone with a smartphone and connectivity to download the app. The app may be especially of interest to young and middle-aged adults in underserved populations who are enthusiastic about maintaining their health but have limited or no access to BP cuff devices. Such users could readily take action upon learning that their usual PP is high through regular app usage. For example, even if procuring medication is difficult, they could still mitigate their condition by eating a healthy diet, maintaining normal body weight, increasing physical activity, and avoiding excessive alcohol^27^. Ultimately, the smartphone PP app could help reduce the massive burden of systolic hypertension in underserved populations and thus global health inequalities.

## Methods

### Development of pulse pressure (PP) computation algorithm

We developed an algorithm to compute PP from the thumb oscillometric measurements, as shown in Fig. 5. The blood volume oscillations during the hand raise are first detected as described in^15^ to yield their peak-to-peak amplitudes and interval lengths as well as the corresponding average ρgh values. The oscillation amplitudes are then plotted against the ρgh values to yield a discrete (shifted) oscillogram. An unweighted moving average filter (5 point) is applied to this oscillogram. A five-parameter function (see Fig. 5) is next fitted to the smoothed discrete oscillogram via nonlinear least squares to yield a continuous oscillogram^19^. PP is computed as the width of the continuous oscillogram between its maximum slope and minimum slope^18^.

### Evaluation of the smartphone PP application

We evaluated the smartphone PP app for usability and accuracy in volunteers under a protocol approved by, and in accordance with the relevant guidelines and regulations of, the University of Pittsburgh IRB (STUDY20060267, 2020 –). We recruited the volunteers amongst students, staff, and local residents. The inclusion criteria were: (i) ages 21 to 65 years; (ii) no cardiovascular disorders (e.g., arrhythmias) other than hypertension; and (iii) no problems with fine motor control. We obtained written, informed consent from 24 participants. Eight of the participants were experienced users; another eight participants were semi-experienced users in that they tried a different version of the app four months earlier; and the remaining eight participants were new users. We did not exclude any of the participants from the study.

**Figure 6:**
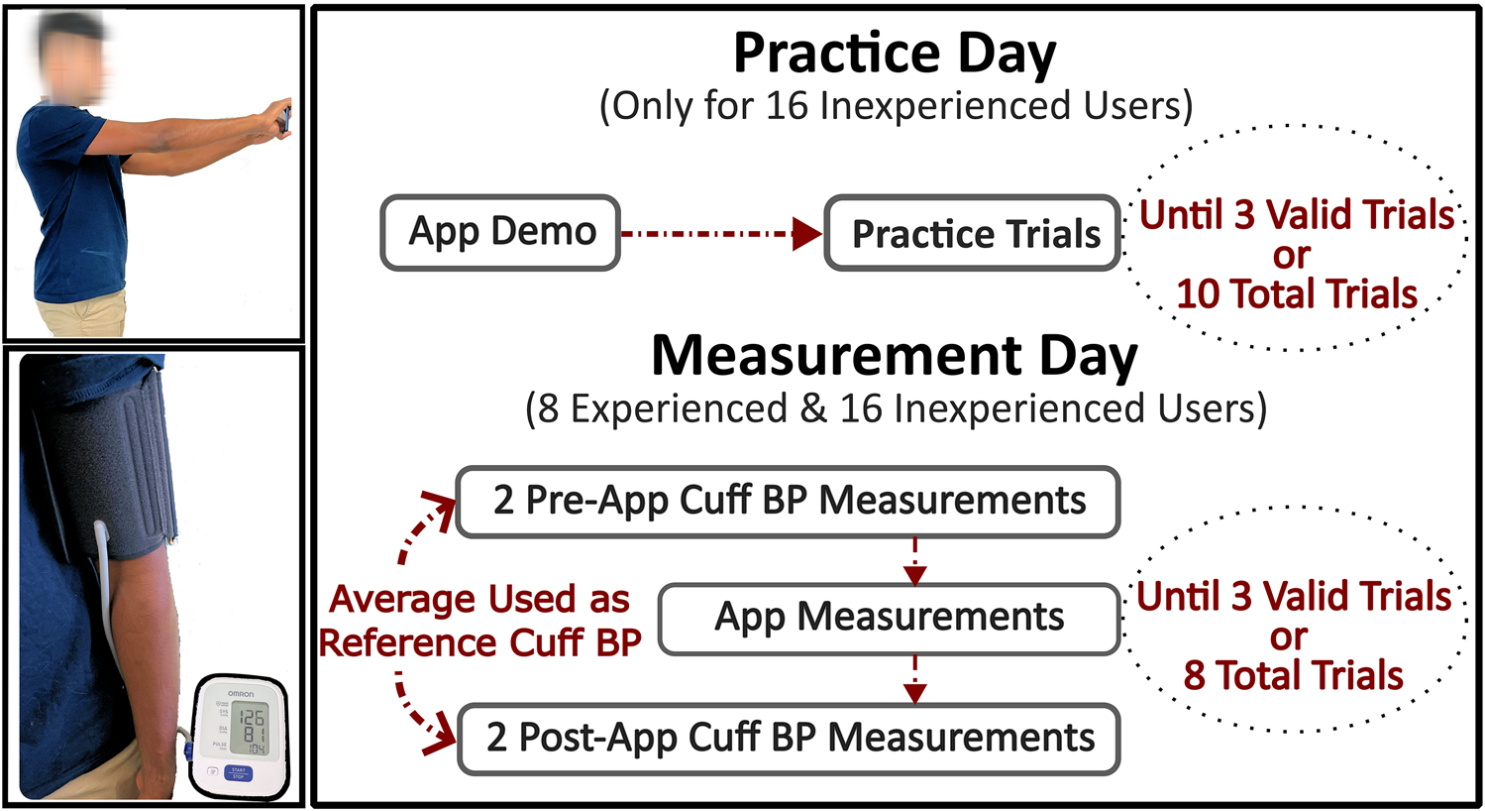
Study protocol to test usability and accuracy of the smartphone PP app. Photographs of the smartphone app and reference automatic cuff device (left). Inexperienced users first learned how to use the app on a practice day (upper right). These users and experienced users then made a series of measurements with the app and reference device on a measurement day (lower right). The subject in the figure provided informed consent to publish the images.

We obtained demographic information and measured the arm length of the participants and then conducted the study protocol shown in Fig. 6. The 16 relatively inexperienced users participated on a practice day. We began by explaining and demonstrating how to use the app. The users then performed practice trials with the app until either three valid measurements were made or 10 total measurements were made. We inspected the app measurements after each trial to assess validity. We defined a valid measurement as (i) a change in the thumb contact area during the hand raise of < 5%; (ii) an oscillogram with amplitudes < 50% of the maximum amplitude on both its increasing and decreasing limbs; and (iii) a visually linear ρgh change. All 24 users then participated on a measurement day. We first obtained two reference BP measurements with a validated automatic arm cuff device (BP7100, Omron). The users then obtained measurements with the app until three valid measurements were made or eight total measurements were made. We then obtained two more reference cuff BP measurements. Two of the experienced users also performed mental arithmetic (randomly pick a 3-digit number and then keep subtracting 17) throughout a single smartphone PP measurement and a single reference cuff measurement to raise their PP. On both days, we allowed > 2 min for rest between each of the measurements and use of a heating pad to keep the thumb warm.

We evaluated app usability by recording the success rate in making valid measurements on the practice day and the measurement day. We evaluated app accuracy against the reference cuff BP values on the measurement day. We applied the PP computation algorithm to the app measurements offline in MATLAB while blinded to the reference measurements. The reason for this offline analysis was that the Github functions that we used in our Android implementation to compute oscillograms and resultant PP were not quite as effective as MATLAB functions. We averaged the multiple smartphone app PP measurements and multiple reference arm cuff PP measurements for each user. We then compared the smartphone app PP to the reference PP using correlation and Bland–Altman analyses. We found that the performance of the new and semi-experienced users were similar and lumped these users together as inexperienced users.

### Analysis of systolic hypertension detection based on PP

We analyzed the National Health and Nutrition Examination Survey (NHANES) database to determine the value of PP in systolic hypertension detection. This de-identified population-level database includes triplicate auscultatory cuff BP measurements, HR, demographics, body measures, smoking history, and clinical conditions from thousands of people in the general population^16^. We combined the NHANES database from 2007–2008 to 2015–2016. We included the average BP, HR, demographics (age, sex, race, and body mass index), and smoking history in our analysis. We excluded participants with missing information and above 65 years old, as our target population for the app is adults less than 65 years old.

We used 70% of the data to train three neural networks for detecting systolic hypertension (systolic BP ≥ 130 mmHg) from average PP alone, only demographics and smoking history, and average PP, HR, demographics, and smoking history. For each network, we used an architecture based on a multilayer Perceptron with a single hidden layer of 10 neurons with rectified-linear activation function and a SoftMax activation function at the output layer. We employed error back propagation with the Limited Memory Broyden–Fletcher–Goldfarb–Shannon algorithm to minimize the cross-entropy between the network output and the actual normotension/systolic hypertension class. We used step size tolerance as a stop criterion for tuning the neural network weights. We used the remaining 30% of the data to test the neural networks in detecting systolic hypertension using standard receiver operating characteristic (ROC) curve analysis. We also likewise trained and tested the neural networks for detecting general hypertension (SP ≥ 130 mmHg or DP ≥ 80 mmHg). We additionally assessed the impact of measurement error on the hypertension detection.

### Supplementary Information

Supplementary Information 1.

Supplementary Video 1.

Supplementary Information 2.

Supplementary Information 3.

Supplementary Information 4.

Supplementary Information 5.

Supplementary Information 6.

Supplementary Information 7.

## Data Availability

The data and code described in this manuscript may be made available upon reasonable request to R.M. (rmukkamala@pitt.edu).
