# Supplementary material for: A smartphone application toward detection of systolic hypertension in underserved populations
Source: Sci Rep. 2024 Jul 4;14:15410. doi: 10.1038/s41598-024-65269-w (PMC11224237; doi:10.1038/s41598-024-65269-w)
Supplement: Supplementary file 1 — Supplementary Information 1. [file 41598_2024_65269_MOESM1_ESM.pdf]

Supplementary Materials 1 - A Smartphone Application Toward Detection of Systolic Hypertension in Underserved Populations: Complete Description of Smartphone Android App

We developed an Android 13.0 application ('app') for the Samsung Galaxy S21 to implement the smartphone PP concept (see Fig. 1). The app makes cuff-like measurements from the thumb using built-in sensors, guides the user in performing thumb and hand maneuvers for cuff-like actuation, and computes PP from the measurements using a cuff algorithm.

The app employs the front camera to measure the PPG waveform from the transverse palmar arch artery in the thumb. The screen light is set to bright; all automatic camera settings (white balance, focus, and exposure) are turned off; the exposure time and frame duration are fixed at 1/30 sec; and the camera sensitivity is maximized. The app uses the z-axis channel of the accelerometer to determine the hydrostatic pressure change, ρgh, from the hand maneuver and the screen touch sensor to measure the x-coordinate of the touch centroid to represent the thumb contact area. The app simultaneously acquires all signals at a sampling rate of 30 Hz. It spatially averages and bandpass filters (0.5 to 6 Hz) the red PPG channel to extract the blood volume oscillations; scales the z-axis accelerometer channel (-1 to 1) by the blood density (ρ = 1 g/ml), the user arm length (measured in cm from the shoulder to the wrist), and a 1.33 conversion factor and then lowpass filters (5 Hz) to yield the ρgh signal; and applies unweighted moving average filtering (3 sec window) to the touch x-centroid to obtain the thumb contact area signal.

The app uses the screen to visually guide the user in performing the thumb and hand maneuvers. The app first displays a one-time initialization screen for the user to enter their arm length and thumb for pressing (see Fig. 2A). The app follows with a rectangular box of fixed size on the screen to guide the user in placing their thumb on the front camera and screen (see Fig. 2B). The app then detects the thumb contact and displays a trio of ovals and the processed PPG waveform to guide the user in determining the proper thumb contact area on the phone. Green shaded and black ovals initially appear (see Fig. 2C). The size of the green oval reflects the real-time thumb contact area quadrupled to make the change in the thumb contact area readily apparent to the user. The black oval is the initial target thumb contact area to ensure absence of blood volume oscillations at the outset. Once the user applies a light press so that the green oval is within the black oval, the last 10 sec of the processed PPG waveform appears as a red trace on the green oval (see Fig. 2D). After the user presses to elicit blood volume oscillations (see Fig. 2E), the app continually performs autocorrelation analysis of the last 2 sec segment of the filtered PPG waveform to detect oscillations when the correlation coefficient exceeds a threshold value of 0.4. A red oval is then displayed whose size is 2% larger than the current thumb contact area as the final target (see Fig. 2F). Once the user presses further so that the green oval reaches the red oval, the app replaces the PPG waveform with a timer to guide hand raising over a 20-40 sec period while the green and red ovals guide the user in maintaining the thumb contact area (see Fig. 2G). The app then displays the blood volume oscillations (see Fig. 2H). We empirically selected the sizes of the rectangular box and target ovals by studying volunteers. The red oval size may be least generalizable, but the percentage increase can be readily adjusted with the app.

The app applies an algorithm to compute PP from the measurements (see Fig. 5). The app finally outputs PP and HR via the average interval length (see Fig. 2I).
