# Supplementary material for: A smartphone application toward detection of systolic hypertension in underserved populations
Source: Sci Rep. 2024 Jul 4;14:15410. doi: 10.1038/s41598-024-65269-w (PMC11224237; doi:10.1038/s41598-024-65269-w)
Supplement: Supplementary file 3 — Supplementary Information 2. [file 41598_2024_65269_MOESM3_ESM.pdf]

Supplementary Materials 3 - A Smartphone Application Toward Detection of Systolic Hypertension in Underserved Populations: Ideal Sensors

Introduction

The 'oscillometric hand raising method' has only been demonstrated in four participants for mean blood pressure (BP) measurement [1] and one participant for systolic and diastolic BP measurement [2]. We first employed 'ideal sensors' to determine the feasibility of the method in a more significant human study. If the oscillometric hand raising method cannot work with ideal sensors, then there would be no need to proceed in developing a smartphone application.

Methods

We studied 19 volunteers under IRB approval. As shown in Fig. S3.1, we placed a transmissive-mode infrared photoplethysmography (PPG) clip with a Velcro tightening wrap to measure finger blood volume oscillations and a fluid-filled tube-manometer system to directly measure the hydrostatic pressure change in the finger induced by hand actuation (ρgh, where ρ is the known blood density (near that of water), g is gravity, and h is the vertical distance between the finger and heart). We also used a volume-clamp finger cuff device (Nexfin, BMEYE) to obtain the finger BP waveform and an automatic arm cuff device (BP7100, Omron) to measure arm BP. We displayed the measurements in real-time using a data acquisition system (MP160, Biopac) and PC. We first obtained a BP measurement with the arm cuff device. We then tightened the wrap while the participants held their hand at heart level to elicit large PPG oscillations (near mean finger BP). The participants then continuously raised their fully extended hand from below the hip to overhead levels by rotating only the shoulder joint in the sagittal plane in about 30 sec. The participants repeated the hand raising a total of three times with at least one minute in between. We lastly obtained BP measurements with the arm cuff device and finger cuff device. Three of the participants also performed squatting exercise to increase their PP and then obtained simultaneous hand raising and arm cuff measurements. In addition, six of the participants performed analogous hand lowering, and a few participants performed the hand raising incrementally in steps of 30 degrees. We computed pulse pressure (PP) from the width of the shifted oscillogram constructed from the ideal sensor measurements, as shown in Fig. 5. We averaged multiple PP measurements from each participant.

Results

Fig. S3.2 shows the PP via hand raising versus finger cuff PP and arm cuff PP. Against finger cuff PP, the correlation coefficient (r) was 0.57, and the bias and precision errors (μ and σ) were -14 and 14 mmHg. Against arm cuff PP, r was 0.75, and μ and σ were -2.9 and 7.4 mmHg.

Fig. S3.3 shows PP via hand raising versus arm cuff PP and PP via hand lowering versus arm cuff PP for the same participants. The r, μ, and σ values were similar for both raising and lowering.

Fig. S3.4 shows a representative PPG waveform during hand lowering followed by hand raising. Even though the PP measurements were similar for both actuations, the maximum oscillation amplitude was consistently smaller during hand lowering regardless of order.

Incremental hand raising did not yield reliable enough oscillograms for PP computation (results not shown). The oscillograms were not sufficiently sampled in particular.

Discussion

The oscillogram obtained via hand raising showed consistent inverted U pattern, and its width was indicative of arm cuff PP but not volume-clamp finger cuff PP. Volume-clamp devices are FDA-

cleared for measuring arm BP, but they have never been validated for finger BP measurement due to a lack of a reference measurement. Agreement with arm cuff PP is also most important. Hand raising and hand lowering yielded similar PP despite blood volume oscillation amplitude differences. Although incremental hand raising/lowering can reduce the variability in the actuation, slow, continuous hand raising/lowering yielded superior oscillograms. We concluded that the oscillometric hand raising/lower method for BP monitoring is worthy of pursuit.

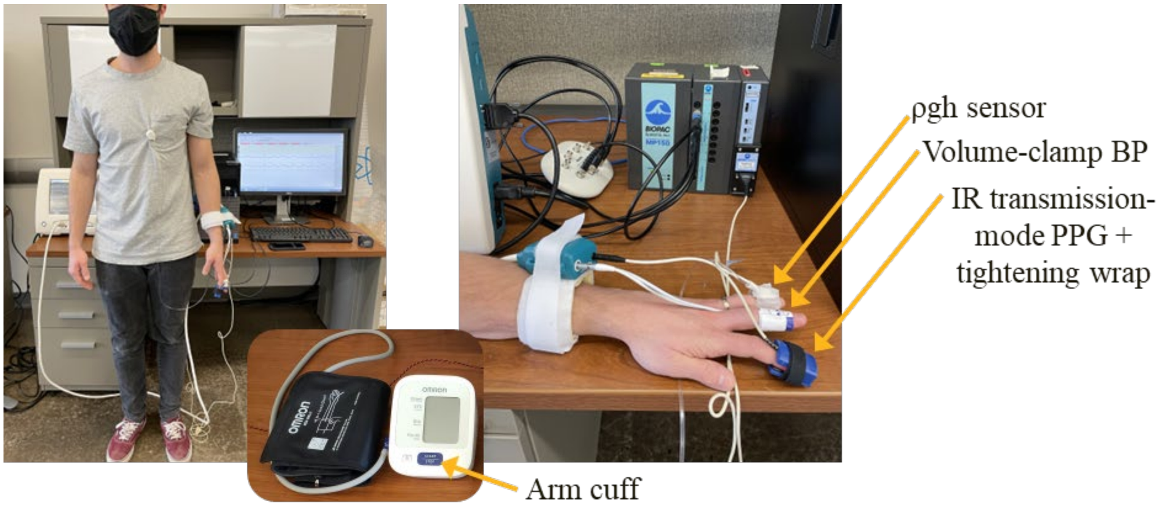

Fig. S3.1. Ideal sensors for recording oscillometric hand raising/lowering measurements. Volume-clamp device for obtaining finger cuff BP. A standard oscillometric device for measuring reference arm cuff BP.

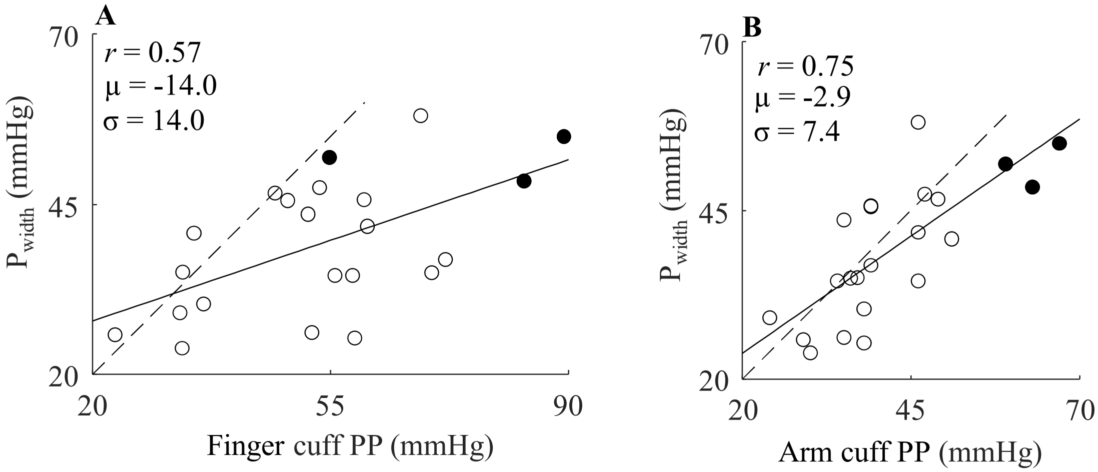

Fig. S3.2. Correlation plots of ideal sensor PP (P_{witdh}) versus **(A)** finger cuff PP and **(B)** arm cuff PP. The unfilled circles are data points from users at rest, whereas filled circles are data points from users after exercise to raise the PP. r, correlation coefficient; μ, bias error (mean of the errors); σ, precision error (SD of the errors); solid line, best line fit; and dashed line, identity line.

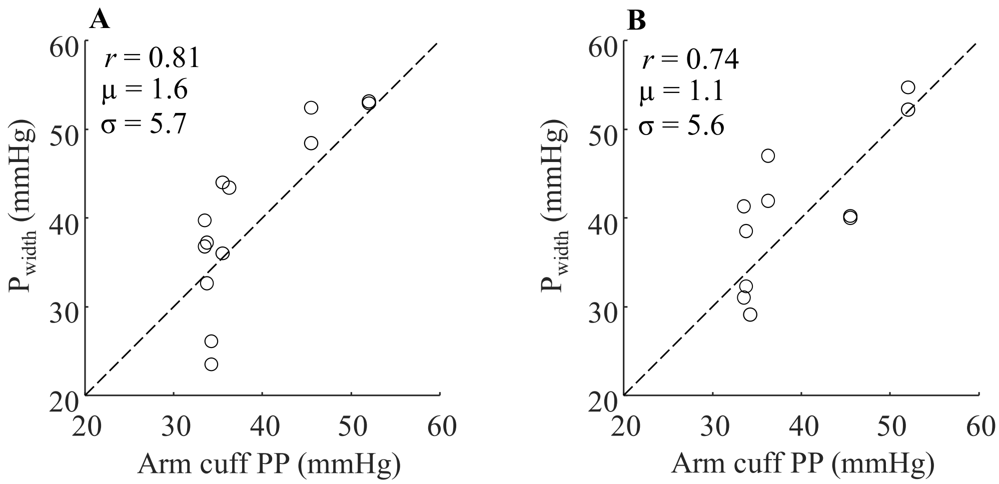

Fig. S3.3. Correlation plots of P_{witdh} versus arm cuff PP for **(A)** hand raising and **(B)** hand lowering.

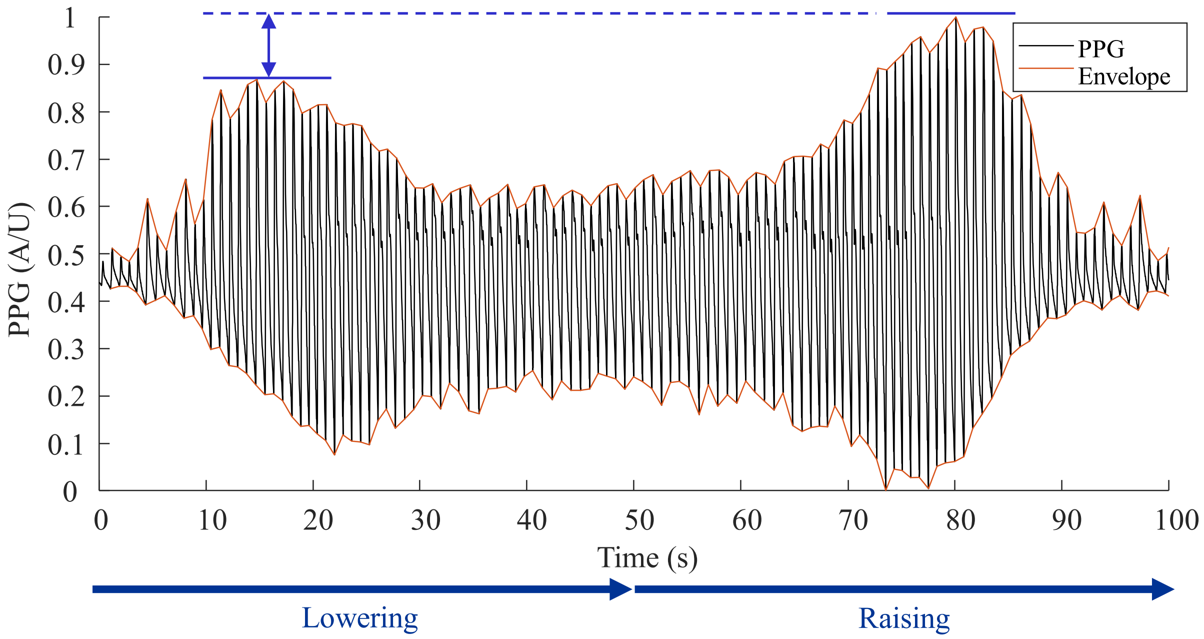

Fig. S3.4. PPG waveform during hand lowering and raising

References

[1] P. A. Shaltis, A. T. Reisner, and H. H. Asada, "Wearable, Cuff-less PPG-Based Blood Pressure Monitor with Novel Height Sensor," in *28th IEEE EMBS Annual International Conference* , 2006.

[2] P. A. Shaltis, A. T. Reisner, and H. H. Asada, "Cuffless Blood Pressure Monitoring Using Hydrostatic Pressure Changes," *IEEE Trans Biomed Eng*, vol. 55, no. 6, pp. 1775–1777, Jun. 2008, doi: 10.1109/TBME.2008.919142.
