# Supplementary material for: A smartphone application toward detection of systolic hypertension in underserved populations
Source: Sci Rep. 2024 Jul 4;14:15410. doi: 10.1038/s41598-024-65269-w (PMC11224237; doi:10.1038/s41598-024-65269-w)
Supplement: Supplementary file 4 — Supplementary Information 3. [file 41598_2024_65269_MOESM4_ESM.pdf]

Supplementary Materials 4 - A Smartphone Application Toward Detection of Systolic Hypertension in Underserved Populations: Wrist Devices

Introduction

Smartwatches and fitness bands currently in use also include sensors to implement our PP measurement concept (see Fig. 1). For those people with access to these devices, the PP measurement would be more convenient in that the contact pressure of the sensor on the skin could be automatically maintained. We thus studied wrist devices without force sensing to measure PP.

Methods

We studied eight volunteers under IRB approval. As shown in Fig. S4.1, we built a Velcro wristband comprising a reflectance-mode infrared PPG sensor to measure blood volume oscillations and the fluid-filled tube-manometer system to directly measure the hydrostatic pressure change, ρgh, induced by hand actuation. We displayed the measurements in real time with the data acquisition system and PC. We positioned the PPG sensor of the wristband over the radial artery. We then attempted to tighten the wristband, but the tightening mechanism was not very effective. For the sake of convenience, we simply instructed the participants to extend their wrist to increase the PPG sensor contact pressure, as shown in Fig. S4.2. The participants raised their arm above the head and extended their wrist to increase the contact pressure until the blood volume oscillations were abolished. The participants then performed hand raising while maintaining their wrist angle. The participants repeated the wrist and hand maneuvers until two complete oscillograms resulted. We obtained BP measurements with the automatic arm cuff device before and after the hand maneuvers and averaged the PP for reference. We computed PP from the wristband measurements, as shown in Fig. 5.

We likewise tested reflectance-mode infrared and green PPG sensors positioned on the back of the wrist. We additionally modified a Samsung Galaxy Watch 3 by incorporating a reflectance-mode infrared PPG sensor therein and attempted to position the PPG sensor over an artery.

Results

Fig. S4.3 shows the PP via the wristband infrared PPG sensor over the radial artery versus arm cuff PP for the seven participants with valid measurements. The r value was 0.93, while the μ and σ values were -0.1 and 5.5 mmHg.

The green PPG sensor on the back of the wrist (similar to consumer devices) yielded very narrow oscillograms indicative of PP in small cutaneous vessels. The infrared PPG sensor on the back of the wrist did not produce clear blood volume oscillations. We could not position the PPG sensor within the smartwatch over the radial or ulnar artery due to the device form factor.

Discussion

We found that PP via the custom wristband with an infrared PPG sensor over the radial artery could be accurate. It appeared more accurate than finger devices likely because the measurement was from the larger radial artery. However, while we believe there is scope to make the wristband tightening mechanism effective, the main problem was that the sensor positioning over the radial artery required an expert and was still extremely difficult and even impossible with a smartwatch form factor. To simplify the positioning, we tried infrared and green PPG sensors on the back of wrist but did not obtain useful oscillograms. We concluded that smartwatches and fitness trackers currently in use cannot be converted into PP sensors.

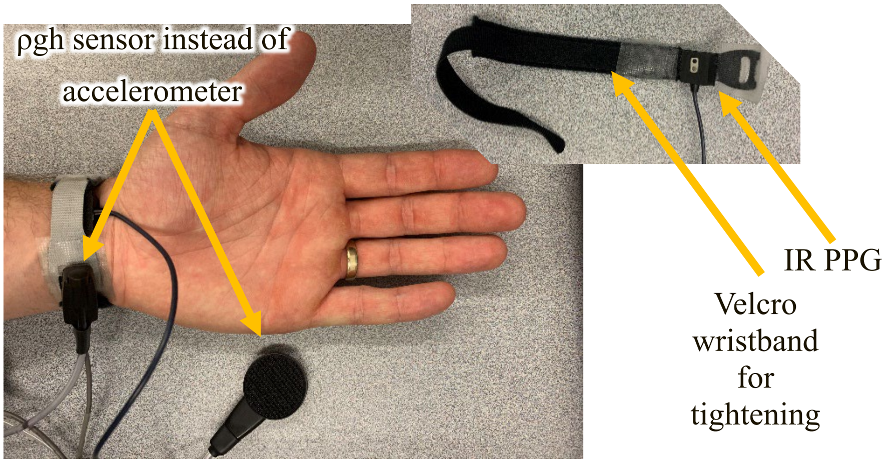

Fig. S4.1. Wrist band for recording oscillometric hand raising/lowering measurements. IR is infrared.

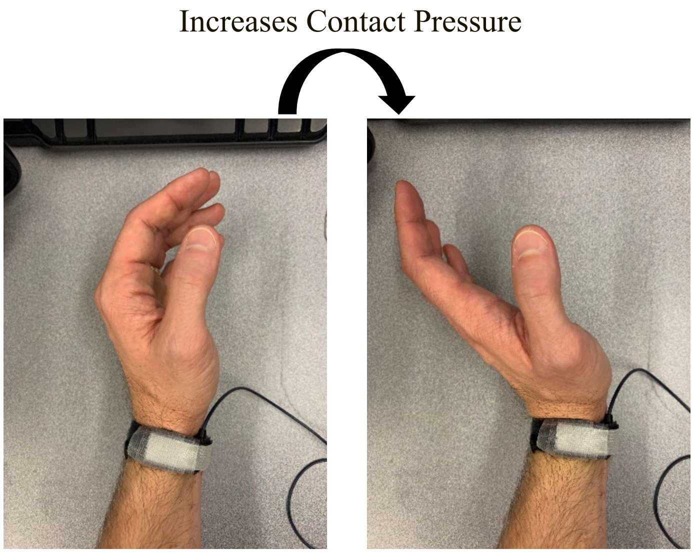

Fig. S4.2. Method for increasing PPG contact pressure with the wrist band. With arm above the head, the wrist is extended to increase contact pressure until PPG oscillations were abolished. The participants then maintain this wrist angle during hand raising.

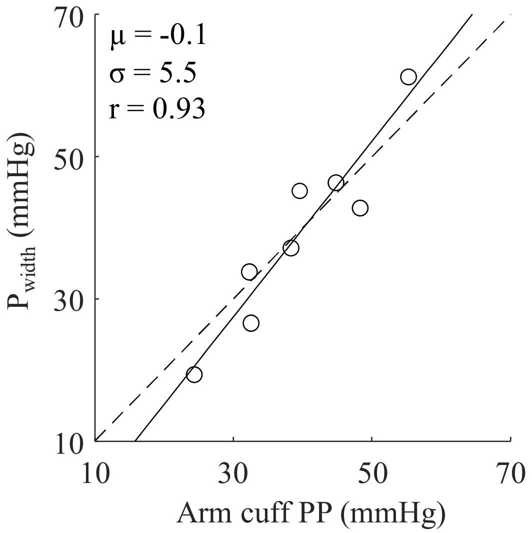

Fig. S4.3. Correlation plot of wrist band PP (P_{witdh}) versus arm cuff PP. r, correlation coefficient; μ, bias error (mean of the errors); σ, precision error (SD of the errors); solid line, best line fit; and dashed line, identity line.
