# Supplementary material for: A smartphone application toward detection of systolic hypertension in underserved populations
Source: Sci Rep. 2024 Jul 4;14:15410. doi: 10.1038/s41598-024-65269-w (PMC11224237; doi:10.1038/s41598-024-65269-w)
Supplement: Supplementary file 5 — Supplementary Information 4. [file 41598_2024_65269_MOESM5_ESM.pdf]

Supplementary Materials 5 - A Smartphone Application Toward Detection of Systolic Hypertension in Underserved Populations: iPhone X App

Introduction

We first investigated our smartphone PP concept (see Fig. 1) using an iPhone X. In contrast to most smartphones, this particular phone includes a sensitive force sensor ('3D touch') under the screen. The phone thus allowed us to assess the feasibility of the user in maintaining the contact pressure on the phone during the hand raising via 'ideal guidance' with a direct force measurement.

Methods

We developed an iPhone XS app, as shown in Figs. S5.1 and S5.2. The app employs the relatively standard front camera to measure a red PPG waveform from the thumb, the z-axis accelerometer channel to determine the hydrostatic pressure, ρgh, induced by hand actuation, and the 3D touch sensor to measure the thumb contact force (CF) in arbitrary units. A user makes a PP measurement with the app in two steps. In the first step (Fig. S5.1), the user places their thumb over the front camera and screen. The app displays a grid on the screen to guide the thumb placement. The user then presses their thumb straight downward to steadily increase the CF while holding the phone at heart level. The app displays the CF in real-time (blue circle) and target lines (red) to guide this thumb pressing. Once arterial occlusion is achieved, the app plots the blood volume oscillations versus the CF, and the CF at maximal oscillation is selected as the target CF. In the second step (Fig. S5.2), the user holds the phone with hands fully lowered and presses their thumb on the front camera and screen to reach the target CF. The app similarly displays the CF in real-time and target lines to guide the thumb pressing. The user then raises their hands steadily with arms straight to all the way above their head over a 20-40 second period while maintaining the target CF. The app continues to display the real-time and target CF for guidance. It thereafter displays the blood volume oscillations versus time during the hand raising for visual inspection. In offline analysis, ρgh is computed by multiplying the z-axis accelerometer value with the blood density ρ and user arm length per basic trigonometry, and PP is computed from the blood volume oscillations and ρgh, as shown in Fig. 5.

We studied the iPhone X app in eight volunteers under IRB approval. We first demonstrated how to use the app. The users were then allowed practice trials. We next obtained BP measurements with the automatic arm cuff device. The users then obtained multiple app measurements with at least one minute between measurements. We concluded with BP measurements with the automatic arm cuff device. One of the participants also performed squatting exercise prior to consecutive app and cuff measurements to increase their PP. We averaged the arm cuff measurements for reference PP.

Results

Fig. S5.3 shows the raw app measurements and constructed (shifted) oscillograms. A valid measurement is first displayed with typical variation in CF during the hand raise (see Fig. S5.3A). The PP computed from this measurement agrees relatively well with arm cuff PP (ssee Fig. S5.3B). An invalid measurement is then displayed with a steady increase in CF during the hand raise (see Fig. S5.3C). This CF increase caused large undeestimation of PP (see Fig. S5.3.d), since the transmural pressure increased due to both hand raising and increasing thumb contact pressure.

Fig. S5.4 shows individual and average PP via the app versus arm cuff PP. The r values were 0.77 and 0.94, and the μ and σ values were -1.1 and -2.1 mmHg and 8.1 and 7.5 mmHg.

Discussion

We observed a general tendency in participants to increase their thumb contact pressure while raising their hands. Although not shown here, we also observed this tendency when volunteers performed the hand raising blindly without any visual guidance of the thumb contact and the opposite tendency when volunteers performed hand lowering. We reasoned that these tendencies are due to the weight of the phone. However, we importantly discovered that this tendency could be readily overcome by instructing the user to hold the phone firmly with the supporting hand and focus on the visual CF feedback. We thus concluded that users could maintain thumb contact pressure on the phone with visual guidance during hand actuation.

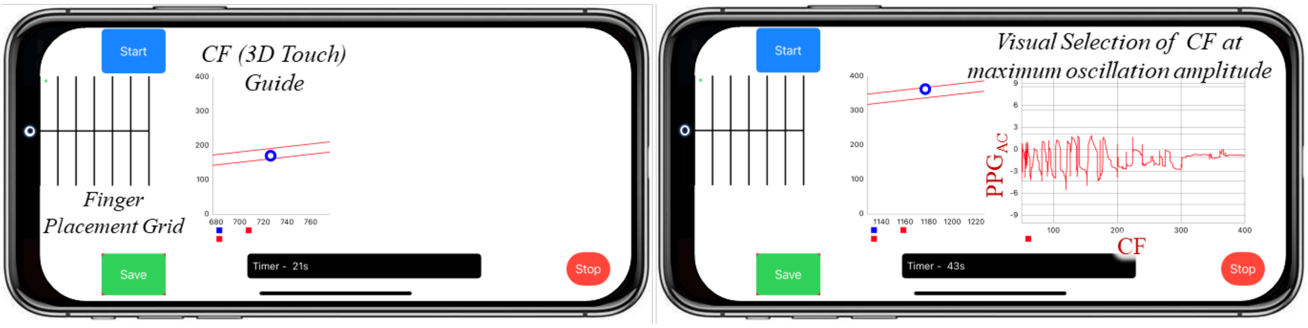

Fig. S5.1. iPhone X app to find optimal contact force (CF) (step 1). The app displays ramps of thumb CF to guide the user to press linearly, and the target CF is determined at the point of maximum oscillation amplitude.

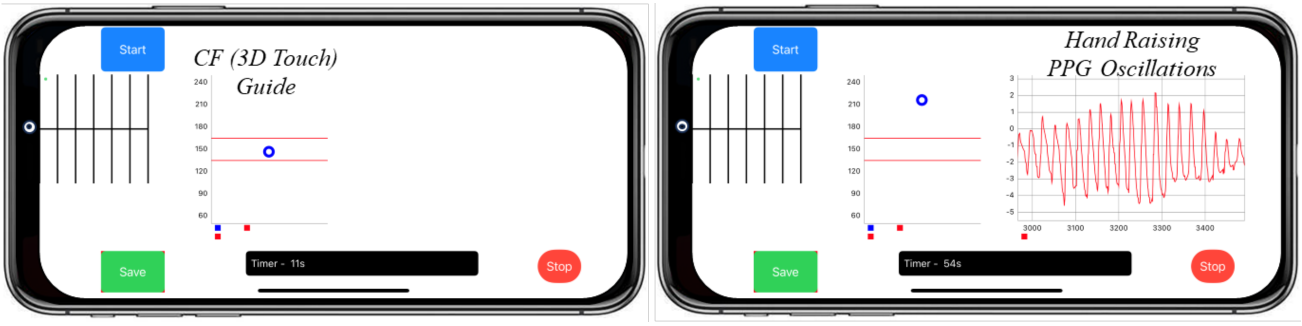

Fig. S5.2. iPhone X app to measure pulse pressure (PP) via hand raising (step 2). The user maintains the blue circle (real-time CF) within the reference guides (red lines) while performing hand raising within 20-40 seconds. The app finally displays the measured PPG oscillations.

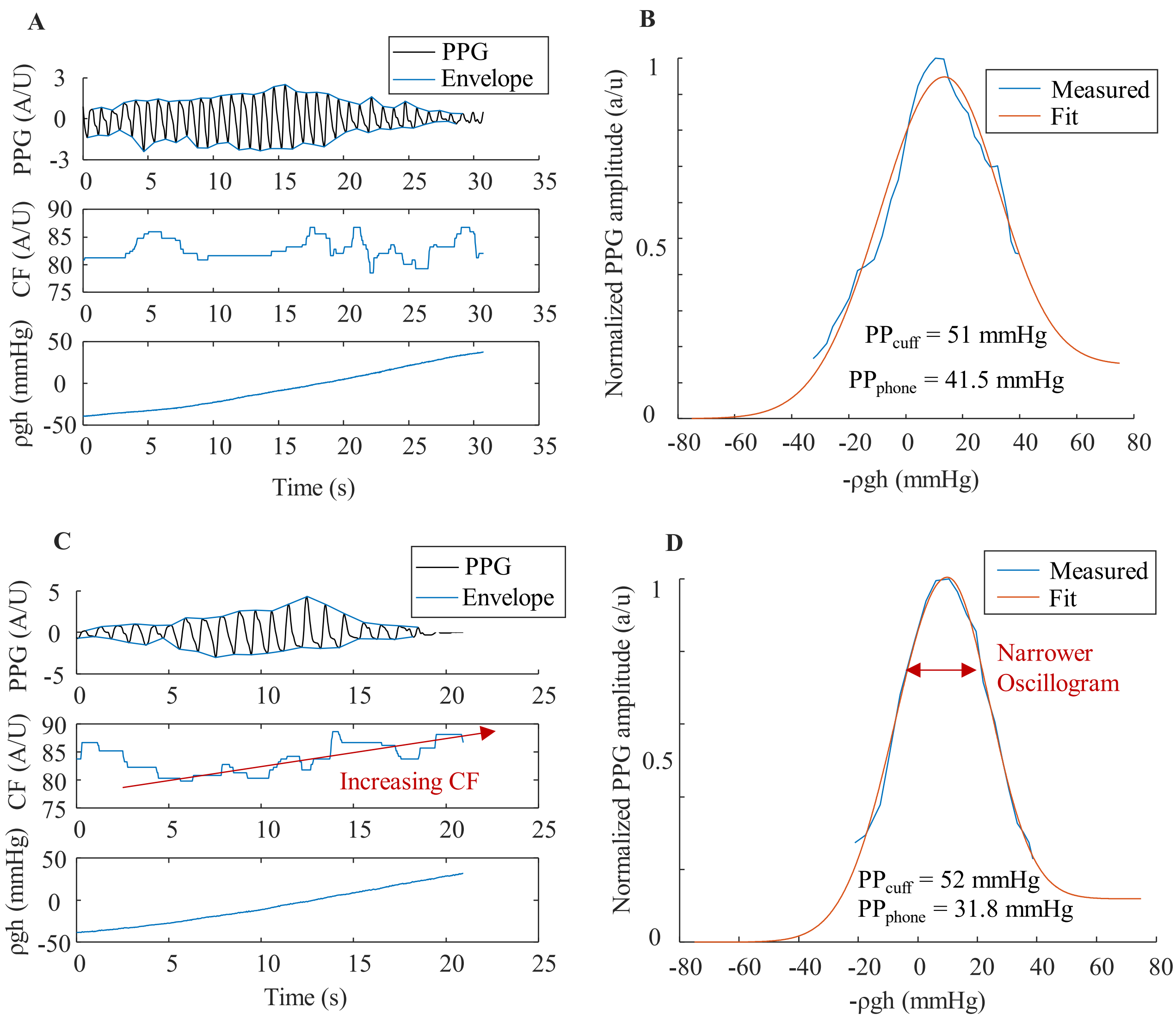

Fig. S5.3. (A, C) iPhone X app measurements for two trials. **(B, D)** Respective shifted oscillograms along with phone and cuff PP.

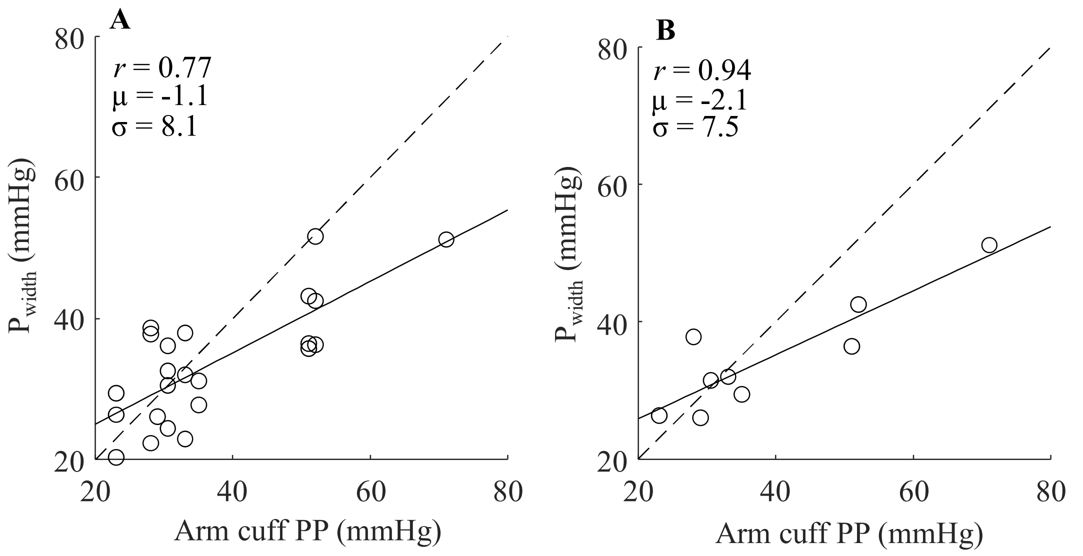

Fig. S5.4. Correlation plots of app PP (P_{witdh}) versus arm cuff PP. **(A)** Three best measurements and **(B)** average of three. r, correlation coefficient; μ, bias error (mean of the errors); σ, precision error (SD of the errors); solid line, best line fit; and dashed line, identity line.
