# Supplementary material for: A smartphone application toward detection of systolic hypertension in underserved populations
Source: Sci Rep. 2024 Jul 4;14:15410. doi: 10.1038/s41598-024-65269-w (PMC11224237; doi:10.1038/s41598-024-65269-w)
Supplement: Supplementary file 6 — Supplementary Information 5. [file 41598_2024_65269_MOESM6_ESM.pdf]

Supplementary Material 6 - A Smartphone Application Toward Detection of Systolic Hypertension in Underserved Populations: Screen Touch-Contact Pressure Relation

Introduction

While most smartphones do not include force sensing, they all include a screen touch sensor to determine where the user is contacting the display (e.g., which app the user wants to open). We studied the screen touch sensor as a surrogate for a force sensor in guiding the determination and maintenance of the proper thumb contact pressure in our smartphone PP concept (see Fig. 1).

Methods

We developed the system shown in Fig. S6.1 to simultaneously measure the screen touch parameters of the Samsung Galaxy S21 smartphone and the contact pressure. A force sensor (Tekscan Flexiforce A201 (4N)) is attached to the back of the phone and specifically under the front camera to measure the reaction force on the index/middle finger during increasing thumb contact pressure (Fig. S6.1AB). A load concentrator of known diameter is used on top of the force sensor to ensure targeted finger force application (Fig. S6.1A). To ensure that the index/middle finger and thumb are experiencing equal forces, the phone is placed on a holder with a pivot (Fig. S6.1C). The measured force is divided by the thumb contact area output by the screen (in display pixels converted to mm) to yield the thumb contact pressure. The smartphone runs an app to acquire the four touch parameters (major and minor radii and x- and y-centroids) during the application of thumb contact pressure on the front camera and adjacent screen. The app also displays one touch parameter in real-time to guide the user in increasing their thumb contact pressure (Fig. S6.1D).

We studied the system in eight volunteers under IRB approval. The volunteers placed their thumb on the front camera and screen and carefully positioned their index/middle finger on top of the force sensor. They increased the contact pressure with app guidance and completed the trial once they could not further increase the pressure. The volunteers performed four to five trials. We used the trial data with the steadiest press for analysis.

Results

Fig. S6.2 shows plots of the touch x-centroid (Tx) versus the thumb contact pressure for each volunteer. The datapoints generally showed an exponential relationship of the form: $Tx = (Tx_{max} - Tx_0)*(1-\exp(-\alpha P))+Tx_0$. The figure also includes the exponential fits. The model parameters varied significantly amongst the volunteers (Tx_0: 82.6-120 (96.9 ± 13.8) pixel units; Tx_{max}: 135.6-172.9 (155.5 ± 10.7) pixel units; and α: 0.005-0.029 (0.015 ± 0.007)) $mmHg^{-1}$).

Fig. S6.3 shows that corresponding plots of the thumb contact pressure versus the touch major radius were similar. However, on average, the decay rate α for the touch x-centroid is 22% smaller than for the touch major radius. The touch x-centroid thus offers greater sensitivity at higher contact pressure.

Fig. S6.4 shows an example of the touch major and minor radii and the touch x-centroid while the thumb contact pressure is kept constant. The touch radii show spurious variations, whereas the touch x-centroid is constant.

Discussion

As expected, the relationship between screen touch parameters and contact pressure tends to increase linearly and then plateau. The relationship varies greatly amongst people, so it may not be possible to mathematically convert screen touch parameters to thumb contact pressure even over the

linear range. Importantly, we found that the touch x-centroid may plateau last, while also being most robust, amongst the four screen touch parameters. We concluded that the touch x-centroid represents the thumb contact area best and could potentially serve as a guide for determining and maintaining the proper thumb contact pressure in our smartphone PP concept.

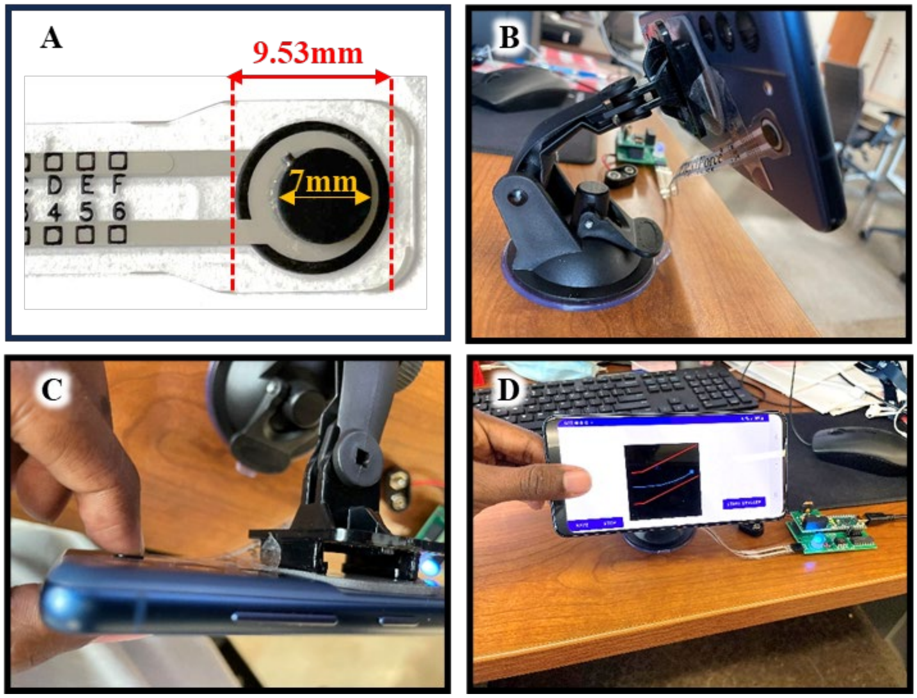

Fig. S6.1. System for testing touch parameter-thumb contact pressure relationship. **(A)** Tekscan force sensor with a sensing diameter of 9.53mm and a load concentrator of 7mm for targeted force application. **(B)** Force sensor affixed to the back of the phone and positioned to be directly under the front camera. **(C)** Users press on the force sensor using the index/ middle finger while pressing on the front camera/phone screen using the thumb. The phone-sensor system is suspended on a free rotating pivot to ensure equal force application on both sides of the phone. **(D)** A smartphone application guides the user to press linearly by showing the touch x-centroid in real-time (blue) and guidelines (red).

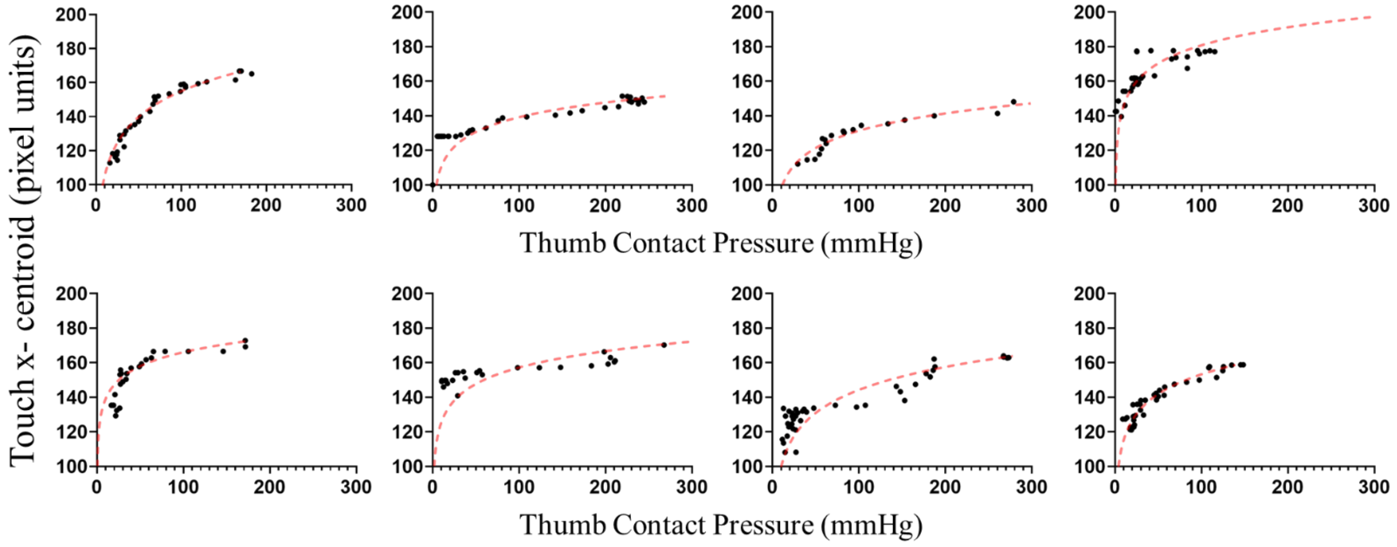

Fig. S6.2. Touch x-centroid position-thumb contact pressure relationship (data points and exponential model fits).

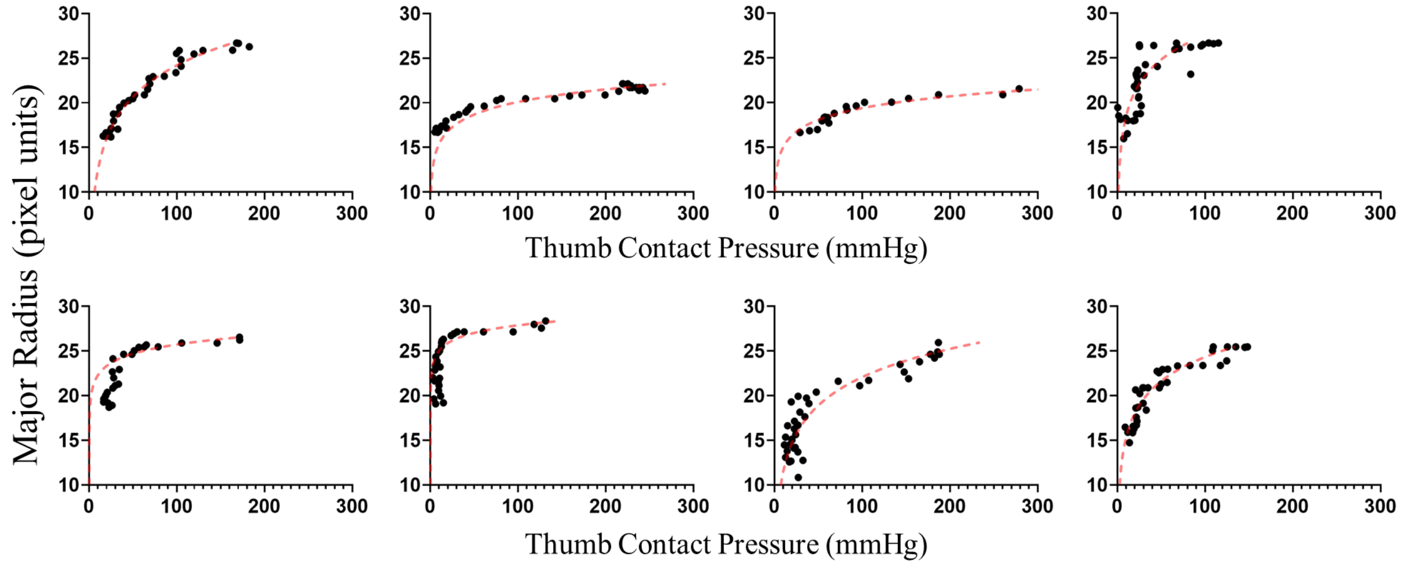

Fig. S6.3. Touch major radius-thumb contact pressure relationship (data points and exponential model fits).

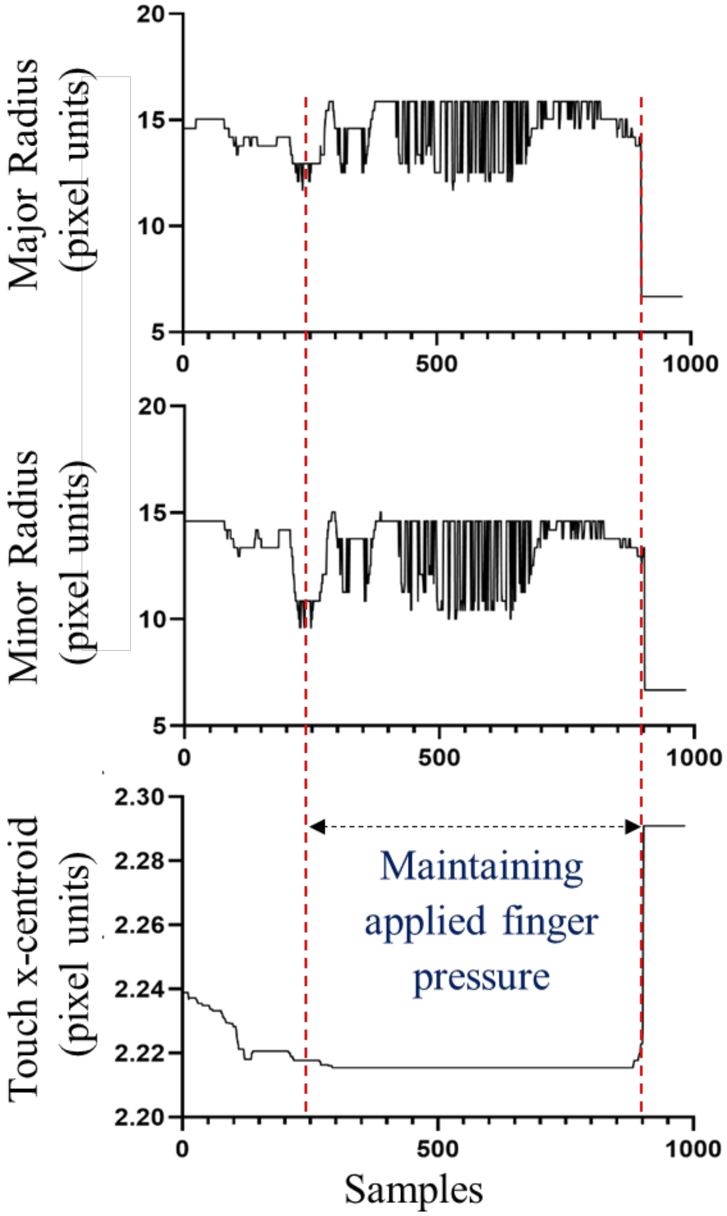

Fig. S6.4. Touch parameter measurements in display pixels during constant thumb contact pressure.
