# Supplementary material for: A smartphone application toward detection of systolic hypertension in underserved populations
Source: Sci Rep. 2024 Jul 4;14:15410. doi: 10.1038/s41598-024-65269-w (PMC11224237; doi:10.1038/s41598-024-65269-w)
Supplement: Supplementary file 7 — Supplementary Information 6. [file 41598_2024_65269_MOESM7_ESM.pdf]

Supplementary Materials 7 - A Smartphone Application Toward Detection of Systolic Hypertension in Underserved Populations: Smartphone Android App 1

Introduction

We developed the first version of an Android app for the Samsung Galaxy S21 to implement our smartphone PP concept (see Fig. 1). Like most smartphones, this phone does not include force sensing. The app is similar to our iPhone X app (Supp. Mat. 5) but uses the screen touch sensor to guide the user in determining and maintaining the proper thumb contact pressure on the phone.

Methods

Fig. S7.1 illustrates the Android app. The app employs the front camera to measure a red PPG waveform from the thumb, the z-axis accelerometer channel along with the user arm length to measure the hydrostatic pressure change, ρgh, induced by hand actuation, and the screen touch sensor to measure the thumb contact area via the touch x-centroid (Supp. Mat. 6). A user makes a PP measurement with the app in two steps. In the first 'initialization' step (Fig. S7.1AB), the user places their thumb over the front camera and screen. The app displays a rectangular box with a grid for different thumb sizes to guide the thumb placement. The user then presses their thumb straight downward to steadily increase the thumb contact area while holding the phone at heart level. The app displays the thumb contact area in real-time and target red lines to guide this thumb pressing. Once arterial occlusion is achieved, the app displays the blood volume oscillations versus the thumb contact area, and the thumb contact area at maximal oscillation is selected as the target area. In the second 'measurement' step (Fig. S7.1C-F), the user holds the phone with hands fully lowered and presses their thumb on the front camera and screen to reach the target thumb contact area (Fig. S7.1C). The app displays the real-time and target thumb contact areas, via green shaded and black circles respectively, to guide the thumb pressing (Fig. S7.1D). The user then raises their hands steadily with arms straight to all the way above their head over a 20-40 second period while maintaining the target area. The app shows a timer and continues to display the real-time and target thumb contact areas for guidance (Fig. S7.1E). The app lastly displays the blood volume oscillations (Fig. S7.1F) and thumb contact area versus time during the hand raising for visual inspection. PP is computed from the blood volume oscillations and ρgh measurements off-line, as shown in Fig. 5. The idea is that the initialization step need only be repeated when inverted U-shape oscillation amplitude patterns are not obtained as a result of large BP variations in the user.

We studied the Android app in 18 volunteers under IRB approval. Ten of participants were experienced users, whereas the other eight participants were new users. We employed a three-day protocol. On the first day, after training, the users performed the initialization step of the app at five different thumb positions. We selected the thumb position for the user as the one yielding the largest maximum blood volume oscillation amplitude. On the second day, after further training, the users practiced the measurement step of the app until three valid measurements were made or 10 total measurements were made. On the third day, the users performed the measurement step until three valid measurements were made or eight total measurements were made. On this day, we also obtained BP measurements with an automatic arm cuff device before and after the app measurements, and one of the participants performed squatting exercise prior to consecutive app and cuff measurements to increase their PP. We allowed at least one minute in between all measurements and averaged multiple arm cuff measurements for reference PP.

Results

Fig. S7.2 shows average PP via valid measurements with the app versus arm cuff PP. The r value was 0.94, and the μ and σ values were -2.0 mmHg and 3.4 mmHg.

Fig. S7.3 shows the success rate in making valid measurements with the app for each user. On average, the success rate was only 30% for inexperienced users and 43% for experienced users. Of the invalid measurements, 46% for inexperienced users and 36% for experienced users were due to an apparent shift in the target thumb contact area. In other words, a complete oscillogram was not obtained despite achieving the target from the initialization step.

Discussion

Selection of the optimal thumb position for each user turned out to be unnecessary, since all central grid positions yielded useful app measurements. One rectangular box size would have been effective for all of the study volunteers. Definition of a personalized rectangular box size, which can be done through a one-time procedure, may only be necessary for people with atypical thumb sizes.

The app could measure PP reasonably well, but it was too difficult to use. The main reason for the low success rate in making valid measurements was that the target thumb contact area was often not suitable for obtaining complete oscillograms and had to be manually adjusted from one trial to the next. The apparent changes in the target could have been due to differences in thumb positioning and pressing angle. The initialization step was also not easy to perform, because the thumb contact area for guidance is not indicative of thumb contact pressure at high pressures. However, the users were able to learn how to perform the measurement step and correctly performed this step 80% of the time on the third day.

We drew three conclusions from this study. First, a standard rectangular box can be used to guide thumb placement. Second, blood volume oscillations, whose amplitude is determined by the contact pressure when the device is at a fixed height, should be used to help guide the determination of the target thumb contact area. Third, the target thumb contact area must be determined for each PP measurement to eliminate variations in thumb positioning and pressing angle.

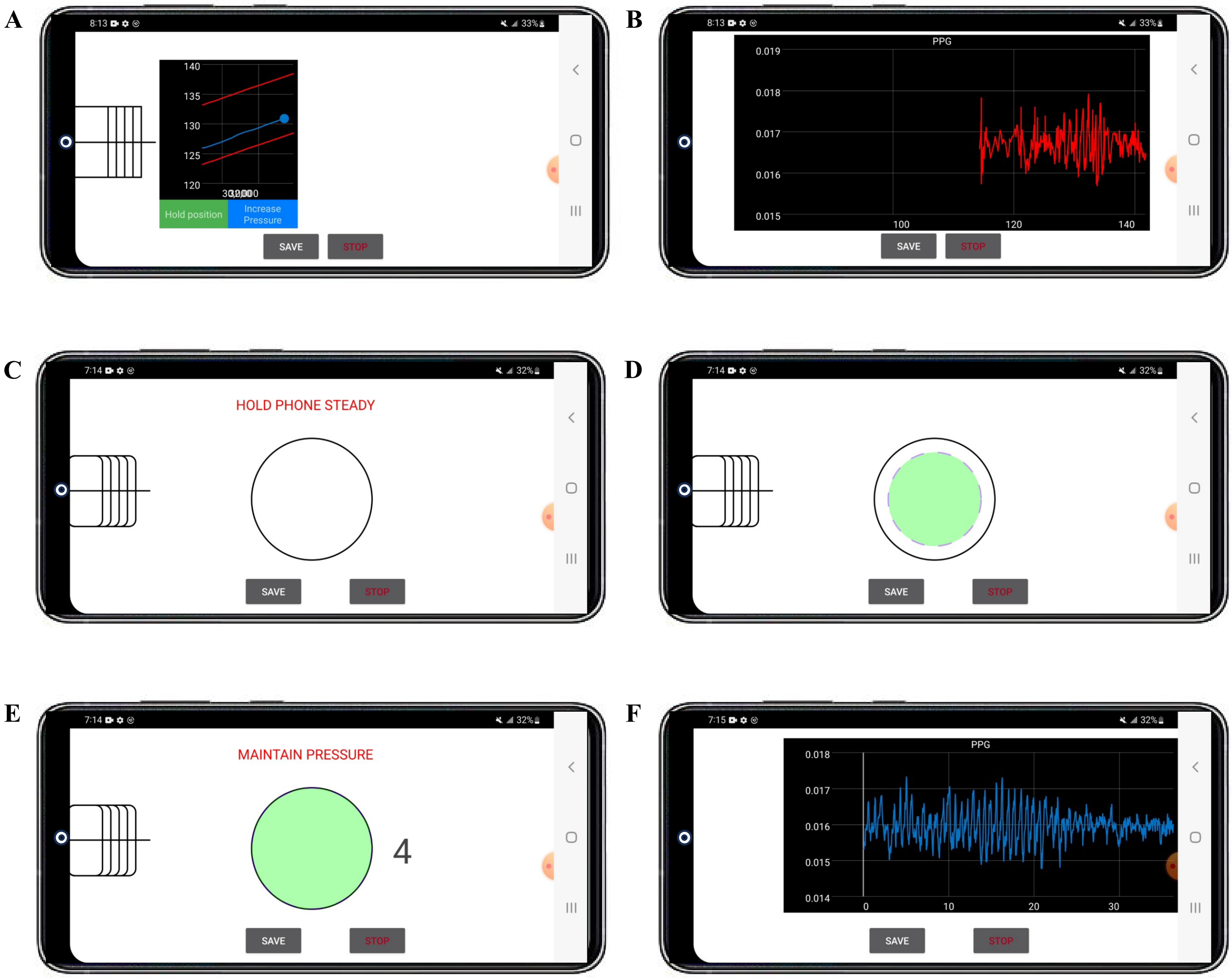

Fig. S7.1. Smartphone Android app (V1) for measuring pulse pressure (PP) via hand raising. **(A, B)** 'Initialization step' to determine optimal thumb placement and target thumb contact area. **(C-F)** 'Measurement step' to achieve the determined target thumb contact area and then perform hand raising within 20-40 seconds.

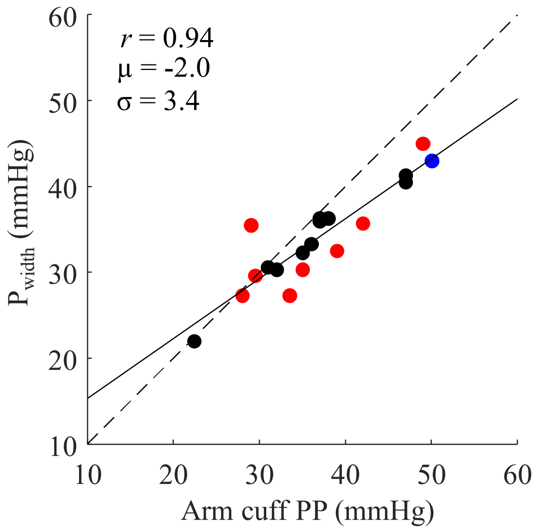

Fig. S7.2. Correlation plot of app PP (P_{witdh}) versus cuff PP. The black dots are data points from experienced users, red dots, from inexperienced users; blue dots, from an experienced user after exercise. r, correlation coefficient; μ, bias error (mean of the errors); σ, precision error (SD of the errors); solid line, best line fit; and dashed line, identity line.

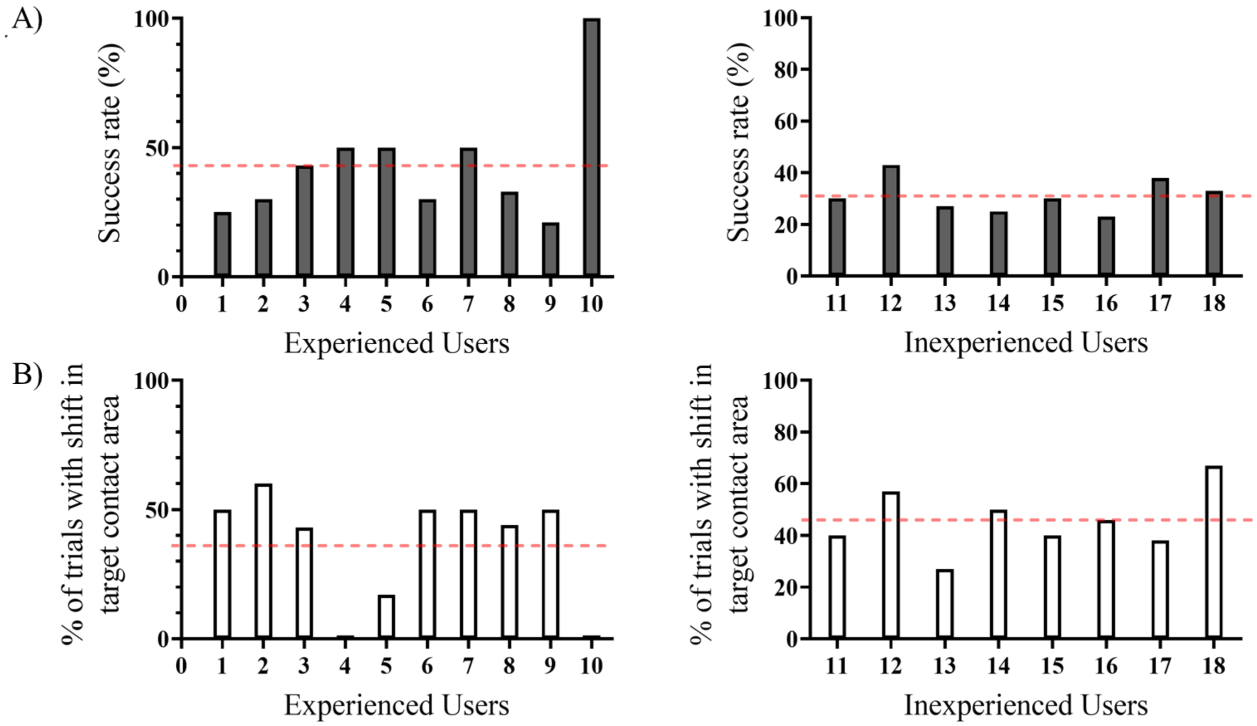

Fig. S7.3. Usability results of the app. **(A)** Success rate to obtain three valid measurements for both experienced and inexperienced users. **(B)** Percentage of invalid measurements due to apparent shift in target thumb contact area for both experienced and inexperienced users. Red dashed horizontal lines are average values.
