# Supplementary material for: A smartphone application toward detection of systolic hypertension in underserved populations
Source: Sci Rep. 2024 Jul 4;14:15410. doi: 10.1038/s41598-024-65269-w (PMC11224237; doi:10.1038/s41598-024-65269-w)
Supplement: Supplementary file 8 — Supplementary Information 7. [file 41598_2024_65269_MOESM8_ESM.pdf]

Supplementary Materials 8 - A Smartphone Application Toward Detection of Systolic Hypertension in Underserved Populations: Smartphone Android App 2

Introduction

We developed a second version of an Android app for the Samsung Galaxy S21 to implement our smartphone PP concept (see Fig. 1). In this version, the target thumb contact area is determined for each PP measurement. Hand lowering (instead of raising) is also employed to facilitate the use of blood volume oscillations in helping to guide the determination of the target area.

Methods

Fig. S8.1 illustrates the Android app. The app employs the same sensors as the first version of the app (Supp. Mat. 7). A user makes a PP measurement as follows. The user places their thumb on the front camera and screen. The app displays a standard rectangular box to guide the thumb placement (Fig. S8.1A). The user then presses their thumb, while holding the phone above the head, to occlude the artery. The app displays the real-time PPG waveform (Fig. S8.1B) to guide the user in abolishing the blood volume oscillations as well as the real-time thumb contact area in the form of a green shaded oval (Fig. S8.1B). Once arterial occlusion is achieved (Fig. S8.1C), the user steadily lowers their hands with arms straight to the fully lowered position over 20-40 sec while maintaining the thumb contact area. The app detects the device lowering initiated by the user via the accelerometer measurement and displays the current thumb contact area as a target black oval as well as a timer (Fig. S8.1D) to guide the hand and thumb maneuvers. The app lastly displays the blood volume oscillations (Fig. S8.1E) and thumb contact area and ρgh versus time (Fig. S8.1F) during the hand lowering for visual inspection. PP is computed from the blood volume oscillations and ρgh measurements off-line, as shown in Fig. 5.

We studied the Android app in six volunteers under IRB approval. We first explained how to use the app. The users then performed practice trials. We next obtained BP measurements with the automatic arm cuff device. The users thereafter obtained multiple measurements with the app. We finally obtained BP measurements with the cuff device. One of the participants also performed squatting exercise prior to consecutive app and cuff measurements to increase their PP. We allowed at least one minute in between all measurements and averaged multiple arm cuff measurements for reference PP.

Results

Fig. S8.2 shows PP via the app versus arm cuff PP. App PP was significantly lower than, and did not correlate with, reference PP. Fig. S8.3 shows gross underestimation of PP by the app despite apparently perfect maintenance of the thumb contact area.

Discussion

The users could achieve arterial occlusion easily, but the PP measurements were substantially underestimated. According to our earlier studies (Supp. Mat. 3 and Supp. Mat. 5), hand lowering should yield similar results to hand raising but users tend to increase contact pressure when raising and decrease contact pressure when lowering due to the weight of the phone. Furthermore, we validated the thumb contact area measurement during only increasing contact pressure (Supp. Mat. 6). We reasoned that the thumb contact pressure must have decreased during hand lowering but was not picked up by the thumb contact area measurement. The likely cause is thumb tissue viscoelasticity (i.e., thumb contact area decreases slowly to a step decrease in thumb contact pressure). We concluded

that thumb contact area is only a useful guide for hand raising in which the tendency is to increase the contact pressure.

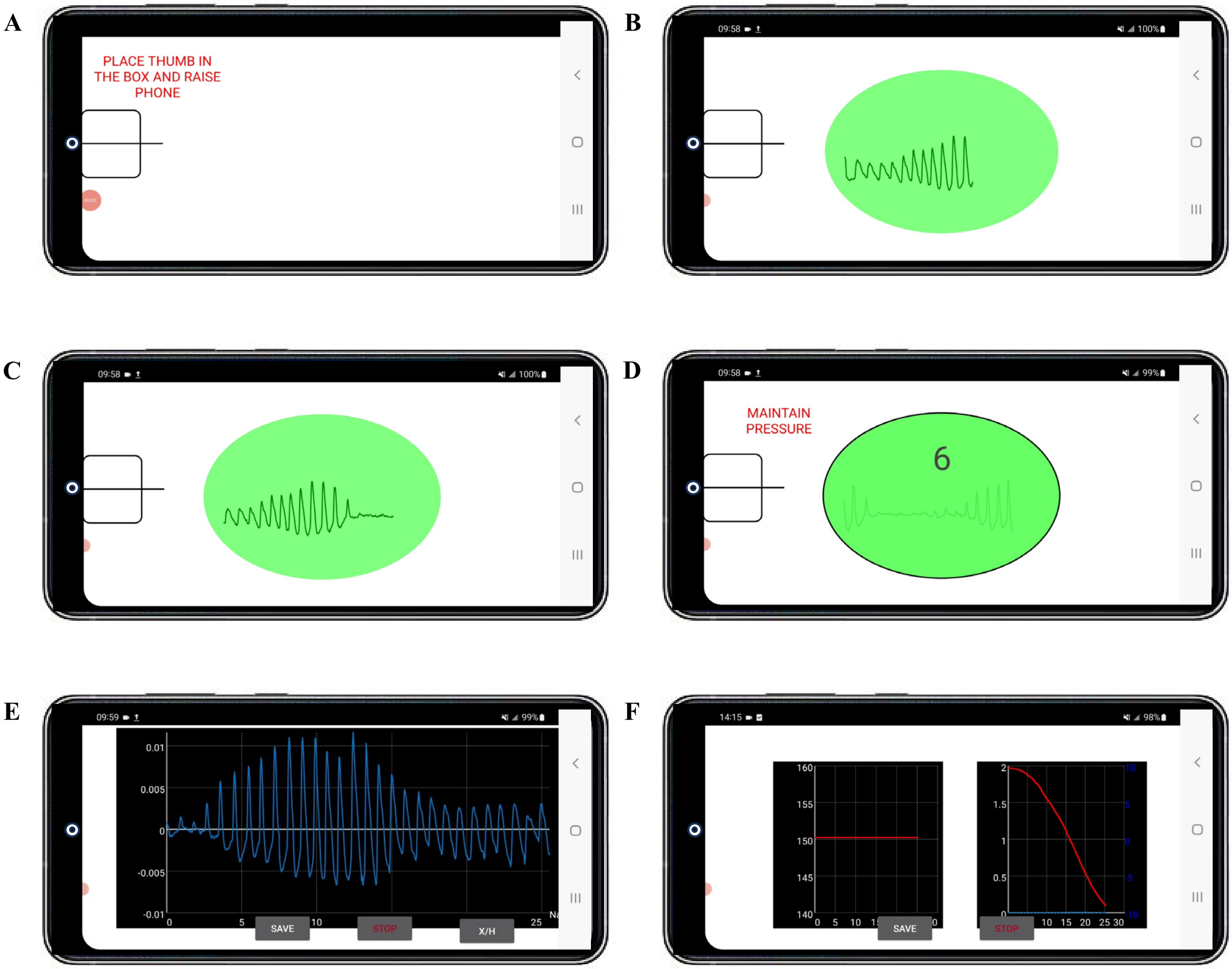

Fig. S8.1. Smartphone Android app (V2) for measuring pulse pressure (PP) via hand lowering. **(A)** The user places their thumb on the front camera/screen and then raises their arm above the head. **(B, C)** The user then presses slowly until they completely occlude the PPG oscillations. **(D)** The user then performs hand lowering within 20-40 secs guided by a timer. **(E, F)** The app finally displays the PPG oscillations, thumb contact area, and ρgh measurement.

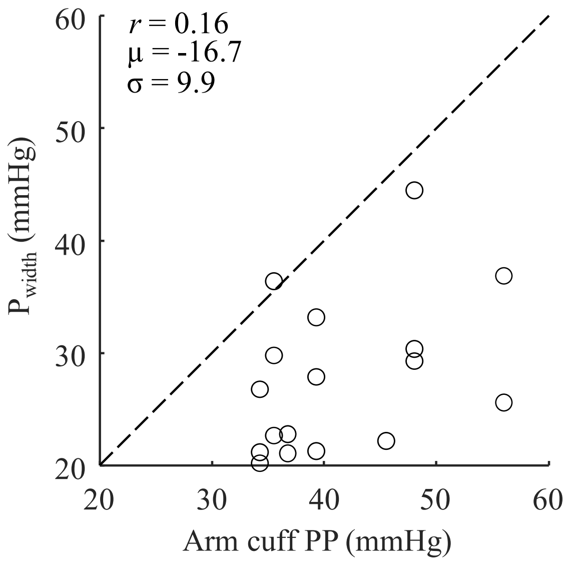

Fig. S8.2. Correlation plot of app PP (P_{witdh}) versus arm cuff PP for the three best measurements. r, correlation coefficient; μ, bias error (mean of the errors); σ, precision error (SD of the errors); and dashed line, identity line.

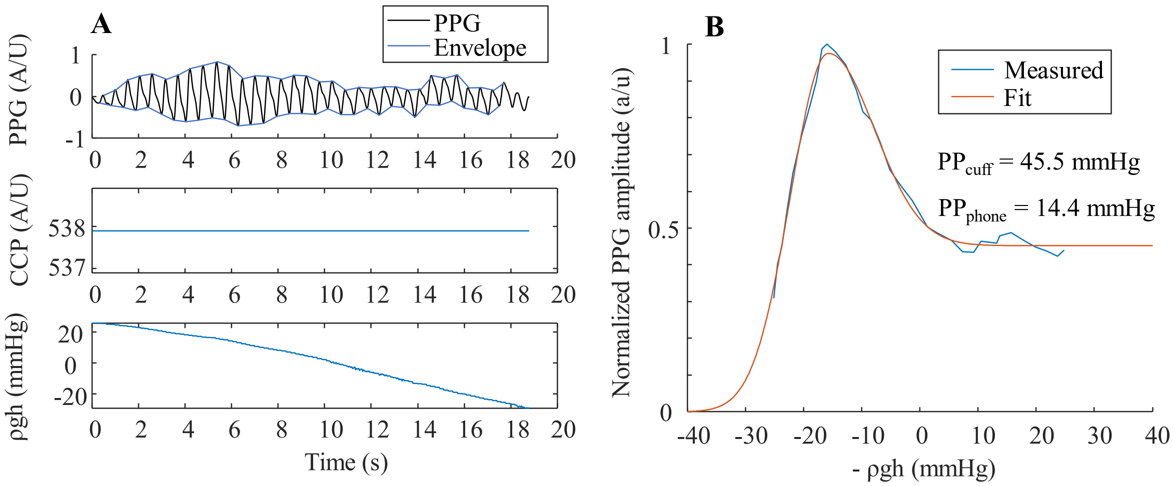

Fig. S8.3. (A) Smarphtone Android app measurements for a trial. CCP corresponds to thumb contact area. **(B)** Respective shifted oscillogram along with phone and cuff PP.
